# Pericentromere clustering in *Tradescantia* section *Rhoeo* involves self-associations of AT- and GC-rich heterochromatin fractions, is developmentally regulated, and increases during differentiation

**DOI:** 10.1007/s00412-020-00740-x

**Published:** 2020-07-17

**Authors:** Hieronim Golczyk, Arleta Limanówka, Anna Uchman-Książek

**Affiliations:** 1 Department of Molecular Biology, Institute of Biological Sciences, John Paul II Catholic University of Lublin, Konstantynów 1i, 20-708 Lublin, Poland; 2 Department of Plant Cytology and Embryology, Institute of Botany, Jagiellonian University, Grodzka 52, 31-044 Cracow, Poland

**Keywords:** Chromocenters, Interphase, Meiosis, Pericentromere, *Rhoeo*, *Tradescantia spathacea*

## Abstract

A spectacular but poorly recognized nuclear repatterning is the association of heterochromatic domains during interphase. Using base-specific fluorescence and extended-depth-of-focus imaging, we show that the association of heterochromatic pericentromeres composed of AT- and GC-rich chromatin occurs on a large scale in cycling meiotic and somatic cells and during development in ring- and bivalent-forming *Tradescantia spathacea* (section *Rhoeo*) varieties. The mean number of pericentromere AT-rich domains per root meristem nucleus was ca. half the expected diploid number in both varieties, suggesting chromosome pairing via (peri)centromeric regions. Indeed, regular pairing of AT-rich domains was observed. The AT- and GC-rich associations in differentiated cells contributed to a significant reduction of the mean number of the corresponding foci per nucleus in relation to root meristem. Within the first 10 mm of the root, the pericentromere attraction was in progress, as if it was an active process and involved both AT- and GC-rich associations. Complying with Rabl arrangement, the pericentromeres preferentially located on one nuclear pole, clustered into diverse configurations. Among them, a strikingly regular one with 5–7 ring-arranged pericentromeric AT-rich domains may be potentially engaged in chromosome positioning during mitosis. The fluorescent pattern of pachytene meiocytes and somatic nuclei suggests the existence of a highly prescribed ring/chain type of chromocenter architecture with side-by-side arranged pericentromeric regions. The dynamics of pericentromere associations together with their non-random location within nuclei was compared with nuclear architecture in other organisms, including the widely explored *Arabidopsis* model.

**Electronic supplementary material:**

The online version of this article (10.1007/s00412-020-00740-x) contains supplementary material, which is available to authorized users.

## Introduction

Spatial arrangement of chromosome domains during interphase is important for biology since it is intimately and functionally linked to gene expression and other essential cellular processes (Kosak et al. 2007; Lanctôt et al. 2007; Schneider and Grosschedl 2007; Fedorova and Zink 2009; Göndör and Ohlsson 2009; Misteli and Soutoglou 2009; Padeken and Heun 2014; Cabianca and Gasser 2016; Poulet et al. 2017; Maass et al. 2018). One of the most striking and spectacular nuclear repatternings is the association of heterochromatic domains—a phenomenon associated with differentiation and development (Ceccarelli et al. 1998 and literature therein; Cerda et al. 1999; Alcobia et al. 2003; Brero et al. 2005; Mayer et al. 2005; Terranova et al. 2005; Gdula et al. 2013; Fujita and Yamashita 2018; Falk et al. 2019). Heterochromatin is a genome part that remains condensed during interphase, detected as deeply staining nuclear bodies—chromocenters, which can associate into higher-order aggregates—collective chromocenters (Heitz 1932; Nagl 1982). Although unclear, the biological function of heterochromatic associations may be multifaceted. Among others, creating nuclear order/constraints, arranging of chromosome territories and nuclear compartmentation, regulating the functional activity of the nucleus, genome guarding and packaging, and meiotic segregation, and facilitating homologous chromosome recognition and juxtaposition have been postulated (Fussell 1987; Dernburg et al. 1996; Ceccarelli et al. 1998; Chubykin 2001; Wijchers et al. 2015; Ostromyshenskii et al. 2018 and literature therein; Jagannathan et al. 2018; Falk et al. 2019).

*Tradescantia spathacea* (Sw.) Stearn (synonyms: *Rhoeo spathacea*, 2n chromosome number = 2x = 12; family *Commelinaceae*) constituting the monotypic section *Rhoeo* of the *Tradescantia* genus is a textbook representative a special genetic system, the so-called permanent translocation heterozygosity (PTH). It is widely accepted that as a PTH species, it had undergone a series of reciprocal translocations as a result of which one complete meiotic ring is formed and there are no two fully homologous chromosomes in the karyotype. Instead, every chromosome is partially homologous to its two neighbors in the ring (Golczyk 2013 and literature therein). The species breeds true for the meiotic ring within which maternal and paternal chromosomes are arranged alternately, establishing two genomes: α and β, each of them consisting of six chromosomes (Golczyk 2013 and literature therein). Since α and β genomes do not recombine and during anaphase I they segregate as whole entities, each of them is viewed as a superlinkage (Ibid.). The perpetuation of the heterozygous condition (αβ) is accomplished by autogamy combined with balanced lethals which eliminate homozygous (αα or ββ) progeny (Ibid.). Although to assemble a complete ring, it is enough that only one of the genomes has been altered by segmental interchanges, more likely is the *Oenothera* scenario, where most or all the members of the karyotype are segmental interchange chromosomes (Cleland 1972; Golczyk 2013 and literature therein).

Interestingly, the pericentromeric heterochromatic regions of this species associate in cycling tissues—both in somatic cells and in prophase I meiocytes (Coleman 1941; Natarajan and Natarajan 1972; Stack and Soulliere 1984; Patankar and Ranjekar 1988; Golczyk and Joachimiak 1999; Golczyk 2011a, b; Golczyk et al. 2005). The ectopic self-adherence of these regions occurs within the highly organized Rabl arrangement, reaching its extreme when binding all the twelve pericentromeres into one large collective chromocenter (Coleman 1941; Natarajan and Natarajan 1972; Stack and Soulliere 1984; Golczyk and Joachimiak 1999; Golczyk 2011a, b), thus resembling the nuclear organization of the *Drosophila* salivary gland nuclei (Zhimulev and Koryakov 2009). While meiotic (peri)centromere associations in the majority of organisms are usually resolved prior to zygotene pairing (Church and Moens 1976; Suzuki et al. 1997; Jin et al. 1998), in *T. spathacea*, they are extremely extensive throughout pachytene and are observed as 1–3 collective chromocenters (Coleman 1941; Natarajan and Natarajan 1972; Golczyk 2011a, b). This unusually high degree of pericentromeric associations was suggested to be directly linked to meiotic ring formation (Coleman 1941; Natarajan and Natarajan 1972). Interestingly, it was previously inferred from the C-banding pattern that the ring-forming variety showed also formation of a ring of chromocenters during root meristem interphase and prophase (Golczyk and Joachimiak 1999, Golczyk unpbl.). However, studying the interphase architecture with the C-banding method on squash preparations is always frustrated by an inability to distinguish between pericentromeric heterochromatin and C-bands located at other chromosomal sites.

Reports on meiotic and mitotic chromocenters in *T. spathacea* were exclusively focused on ring-forming plants. However, there is also a bivalent-forming (six bivalents) variety possessing one of the translocation genomes (β genome) in double dose (Golczyk 2011c). This rare homozygous form is likely derived from a ring-forming individual through occasional breakdown of the balanced lethals (Ibid.). Thus, as long as the heterochromatin behavior of this variety is unknown, a comprehensive view on the pericentromere associations in the section *Rhoeo* is impossible.

Little is known on how extensive and frequent the pericentromere associations in somatic cells of *T. spathacea* are. The limited reports rely on classical absorption staining or simple fluorescence (Huskins and Steinitz 1948; Mosiołek et al. 2005). They are however contradictory and seem to suffer from an inability to trace pericentromeric heterochromatin specifically (see Discussion).

Notably, our results obtained so far show that the two base-specific differential fluorescent techniques can serve to reliably study the pericentromeric associations in the section *Rhoeo*. First of them, DAPI/Actinomycin D (DAPI/AMD) technique which, by binding a non-fluorescent compound (Actinomycin D) to GC-regions reveals highly contrasting DAPI fluorescence of AT-rich chromosome domains (see Schweizer and Ambros 1994), has been recently shown to mark exclusively AT-rich pericentromeric heterochromatin on all chromosomes in the karyotypes of both *T. spathacea* varieties (Golczyk et al. 2010; Golczyk 2011c). Using this method, all heterochromatic pericentromeres can be unambiguously distinguished from other regions on chromosomes and in nuclei (Golczyk et al. 2010; Golczyk 2011a, b, c). The application of another differential technique—Chromomycin A_3_/Distamycin A/DAPI (CMA_3_/DA/DAPI), which specifically stains GC-rich regions and quenches unspecific fluorescence with the use of non-fluorescent Distamycin A (see Schweizer and Ambros 1994)—resulted in a proficient detection of GC-rich chromatin fractions (Golczyk et al. 2010; Golczyk 2011c). The latter are present in each pericentromere as one or two lateral bands flanking the central AT-rich pericentromeric block, but also constitute heterochromatin of nucleolus organizer regions (NORs), which in both varieties are all localized in telomeric positions (Ibid.). Thus, while DAPI/AMD fluorescence marks exclusively pericentromeres, CMA_3_/DA/DAPI signals are present both at pericentromeres and telomeric NOR-heterochromatin.

Since heterochromatic pericentromeres are composed both of AT-rich and GC-rich distinct chromatin domains, a question arises then whether both domain types participate in pericentromeric associations. Another related problem is the internal organization of a pericentromeric collective chromocenter in meiotic and somatic cells. Previous research in the ring-forming *T. spathacea* has revealed the existence of a stage in the pachytene chromocenter development when pericentromeric GC-rich chromatin was located peripherally in relation to the aggregated AT-rich domains, indicating non-random side-by-side positioning of the pericentromeres (Golczyk 2011b). Whether such a spatial relationship between the two distinct and differently composed types of pericentromeric chromatin is an exclusive attribute of the meiotic path leading to ring formation or represents a more universal chromocenter organization remains to be answered.

Here we portray the unusual nuclear architecture of *Tradescantia* section *Rhoeo* with the intention to rediscover it for the scientific community. Specifically, the goal of the present study was to revisit comprehensively the behavior of pericentromeres in cycling cells (mitotic and meiotic) and in differentiated tissues during development—both in the ring-forming variety (meiotic ring of twelve chromosomes) and bivalent-forming variety (six bivalents)—to place the heterochromatin dynamics in a more broad context than ever before. For this purpose, we used base-specific fluorescent techniques: DAPI/AMD and CMA_3_/DA/DAPI staining, which are highly effective for gently squashed thick preparations containing masses of cytoplasm-rich cells with rigid walls (see current Results) known to be impenetrable for molecular probes in FISH procedures. In particular, we used the two methods together with multifocal extended-depth-of-focus (EDF) imaging to (i) assess the number of pericentromeric fluorescent foci and their position to reveal general modes of pericentromere arrangement/behavior within interphase nuclei of cycling and differentiated cells; and (ii) bring to light the involvement of each type of heterochromatin (AT-rich or GC-rich) in associations by assessment of the global level of GC-rich and AT-rich heterochromatic associations and by further exploration of the structure of the pericentromeric chromocenters and the arrangement of their AT- and GC-rich components.

Our results add an entirely new dimension to what has been done so far by giving the first comprehensive description of the extensive association of heterochromatic pericentromeres, which we found to be an inherent universal trait of mitotic and meiotic cycling cells and of differentiated tissues both in ring- and bivalent-forming plants. Based on the ability to distinguish AT- and GC-rich sites, we describe the internal non-random organization of the collective pericentromeric chromocenter and demonstrate the progressive self-attraction of the AT- and GC-rich domains to be involved in the developmentally regulated pericentromere associations. The scenario revealed by us is that the average chromatin dynamics of somatic cycling cells oscillates around 5–6 pericentromeric chromocenters, which means that the formation of pericentromere pairs is a dominant type in the largest fraction of the root meristem nuclei. The association process, however, increases during differentiation, ending up with one chromocenter in terminally differentiated tissues as the ultimate nuclear configuration. We successfully revisit all the unusual signs of the highly prescribed nuclear architecture, which we also compare with interphase chromatin arrangement in other organisms, including the widely explored *Arabidopsis* model.

## Materials and methods

### Plant material

The typical ring-forming (ring of 12 chromosomes) variety known also as *Rhoeo discolor* (Golczyk et al. 2005, 2010) and the bivalent-forming (6 bivalents) variety concolor obtained from Kew Botanical Garden (Golczyk 2011c) were grown in pots filled with soil in a greenhouse at 25–27 °C. Their young ca. 2–4-mm-long flower buds and small leaf fragments were excised and fixed in 3:1 ethanol-glacial acetic acid. To induce rooting, the stems were cut off at their basal nodes and the cuttings were further kept in glass jars filled with tap water and wrapped with aluminum foil. The water was refreshed each day. Vigorously growing ca. 2–3-cm-long adventitious roots were excised and fixed immediately in freshly made 3:1 ethanol-glacial acetic acid for 30 min.

### Cytological techniques

Fixed material was washed in 0.01 M citric buffer pH 4.6–4.8 for 3 × 15 min at room temperature (RT) and then macerated at 37 °C for 30 min in a mixture of 1% (v/v) pectinase (Sigma-Aldrich) and 1% (w/w) cellulase (Sigma-Aldrich) in 0.01 M citric buffer pH 4.6–4.8. The material was washed in the same citric buffer at RT for 3 × 10 min and placed in a drop of citric buffer on a slide. The reference tissues, i.e., pachytene meiocytes, root meristem, root hairs, leaf parenchyma, and epidermis, were isolated under a binocular stereoscopic microscope using fine needles. For monitoring root development, the roots destined for sectioning were divided using a fine razor blade into six 1-mm-thick transverse sections, i.e., five successive sections representing the first five root millimeters and the sixth slice representing the tenth millimeter. After removing the citric buffer from the slides, some amount of 45% acetic acid was dropped onto the fragmented material and covered with a coverslip. The material was gently squashed between the slide and the coverslip, and then the preparations were frozen in liquid nitrogen. The coverslips were removed and the preparations were air-dried. Only freshly made preparations were used for further treatments. To check briefly the quality of the squash technique (e.g., if the material is well spread on the slide), we did a quick and simple non-differential fluorescent staining, by mounting some of the preparations in a drop of Vectashield medium (Vector Laboratories) supplemented with DAPI (4′-6-diamidino-2-phenyloindole, 1 μg/ml). DAPI/AMD and CMA_3_/DA/DAPI techniques were carried out as follows: The preparations were stained with (1) DAPI (Sigma-Aldrich) or (2) chromomycin A_3_ (CMA_3_, Sigma-Aldrich) and counterstained with (1.1) actinomycin D (AMD, Sigma-Aldrich) or (2.1) distamycin A (DA, Serva) followed by (2.2) DAPI, respectively, and mounted in glycerin medium as described by Schweizer and Ambros (1994). Finally, the preparations were sealed with rubber cement (Marabu) and aged at 4 °C for one or several days (DAPI/AMD) or for at least 3 weeks (CMA_3_/DA/DAPI).

### Microscopy

Detailed microscope observations of the nuclear structure were carried out with Nikon Eclipse 80i and Ni-U epifluorescence microscopes under × 100 and × 60 planachromatic immersion objectives. Fluorescence signals were visualized with the aid of two filter sets, each with a narrow-band excitation fitted precisely to the corresponding emission peak of the HBO lamp: (1) excitation 360–370 nm and emission 435–485 nm for DAPI; (2) excitation 430–440 nm and emission 470 nm for CMA_3_. Extended-depth-of-focus (EDF) images were obtained by capturing 10–15 different focal planes of the same object by cooled monochrome DS-2MBWc or DSQi1 cameras (Nikon) both controlled by NIS Elements software (Laboratory Imaging, Ltd.). The frames were stacked and combined into one image using the EDF function.

### Data analysis

A single collection representing one of the 11 studied tissue types (pachytene meiocytes, root meristem, root hairs, leaf parenchyma, leaf epidermis, and 6 root sectors) consisted of 1000 nuclei derived from five plants (200 nuclei from each plant) representing the same variety and subjected to the same type of the fluorescent technique (DAPI/AMD or CMA_3_/DA/DAPI). The mean number of AT-rich or GC-rich domains per nucleus for each collection was calculated. The multiple pairwise comparisons of the cell collections were done with the use of the Kruskal-Wallis ANOVA test at the 0.05 significance level. To facilitate further discussion, we referred an interphase AT-rich domain to as “collective chromocenter” if a nucleus possessed 1–3 such structures (see Introduction). To study the details of chromocenter internal structure, we mainly focused on nuclei with one big collective chromocenter.

## Results

### Pericentromere attraction in cycling tissues: pachytene and root meristem nuclei

**Fig. 1:**
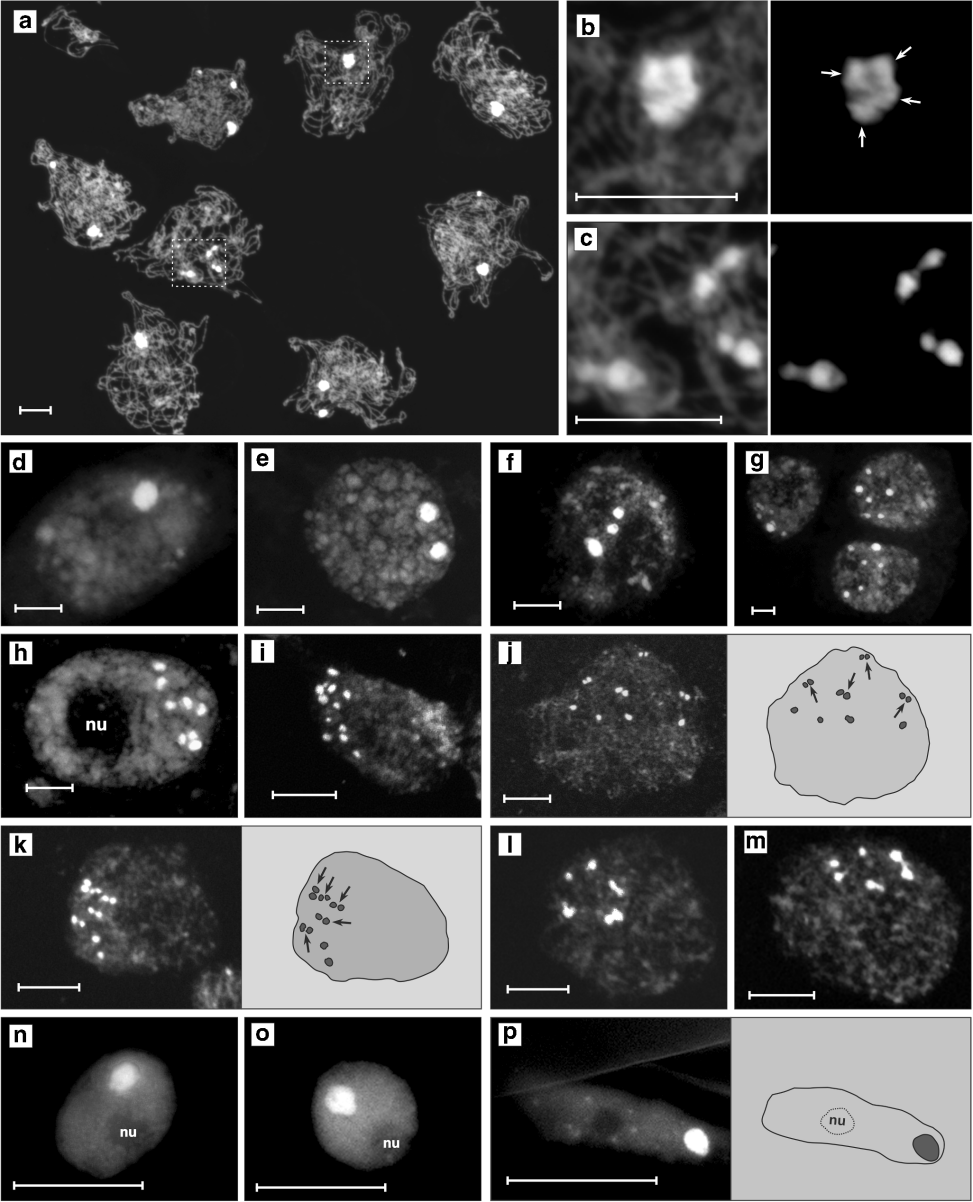
**a**–**p** AT-rich (DAPI/AMD technique) pericentromeric heterochromatic domains of the ring-forming (**a**–**d**, **f**–**g**, **i**, **k**, **m–n**, **p**) and bivalent-forming variety (**e**, **h**, **j**, **l**, **o**) of *T. spathacea*. bars = 10 μm; nu, nucleolus. **a**–**c** Pachytene nuclei and compound structure of their chromocenters; dash lined boxes of **a** are magnified in **b** and **c** with right panels obtained by capturing the same objects using low exposure time settings of the camera; arrows in **b** point to four AT-rich domains; each of the three meiotic chromocenters in **c** is composed of two AT-rich domains. **d**–**m** Nuclei of the root meristem (**e**–**f**, **h**–**m**), 10-mm root sector (**d**) and 1-mm root sector (**g**); arrows in right panels of **j** and **k** point to pericentromere pairs. **n**–**p** Nuclei of leaf parenchyma (**n**), leaf epidermis (**o**), and root hair (**p**)

**Fig. 2:**
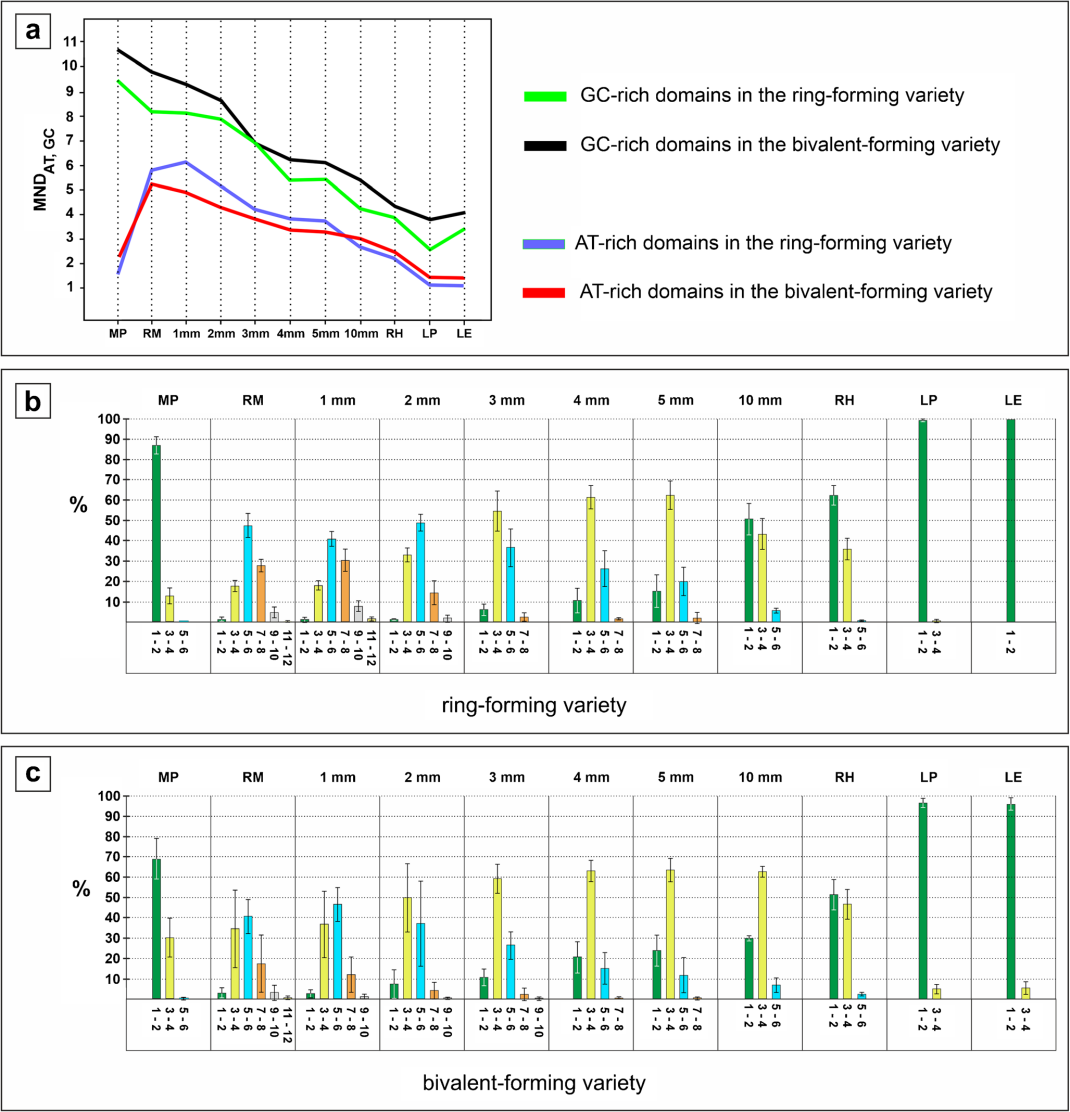
**a**–**c** Changes in basic parameters: mean number of AT- or GC-rich domains per nucleus (**a**) and frequency (%) of the nuclei with a given number of AT-rich domains in the ring-forming (**b**) or bivalent-forming (**c**) variety. MND_AT, GC_, mean number of AT-rich or GC-rich domains per nucleus; MP, meiotic prophase (pachytene); RM, root meristem; 1 mm–5 mm, 10 mm = 1 mm–5 mm, 10 mm root sectors; RH, root hairs; LP, leaf parenchyma; LE, leaf epidermis; 1–2, 3–4, 5–6, etc. = nuclear classes characterized by the presence of 1–2, 3–4, 5–6, etc. domains per nucleus. Vertical bars in **b** and **c** are standard deviations; to render the graph lines visible, standard deviations for **a** are not included. They are given in Table S1

In all the collections of both varieties, the AT-rich pericentromeric domains were clearly delimited from the rest of chromatin as brightly fluorescing foci with a size inversely proportional to their number (Fig. 1a–p). They were typically engaged in diverse polarized associations, i.e., located on one nuclear pole in a small region of a nucleus—at the nuclear border or close to it, and opposite to the terminal nucleolus (e.g., Fig. 1h), thus complying with the Rabl arrangement (Rabl 1885). This polarization is especially well pronounced in meiotic prophase due to an unusually strong tendency of all the meiotic pericentromeric regions to cluster into one or several chromocenters (Fig. 1a; S1a). While the range is 1–5 AT-rich domains per pachytene nucleus, the means are 1.6 and 2.1 for the ring- and bivalent-forming variety, respectively (Fig. 2a, Table S1a). Accordingly, the extreme class of meiotic nuclei with 1–2 AT-rich domains is the prevailing nuclear fraction (Fig. 2b, c) constituting ~ 87% in the ring-forming variety and ~ 69% in the bivalent-forming variety (Table S2a).

In root meristem of the two varieties, the range was markedly broader when compared with meiotic prophase, i.e., 1–12 AT-rich domains per nucleus but both extreme nuclear classes (nuclei with 1–2 or with 11–12 AT-rich domains) were rare (0.3–3.1%). There seemed to be a balance between pericentromere association and dissociation—with 5–6 AT-rich domains occupying the highest peak set up at 41–48% frequency in the middle of the distribution (Fig. 2b, c; Table S2a). Correspondingly, the mean number of AT-rich domains per nucleus was 5.2–5.9 for both varieties (Table S1a).

One of the chromatin configurations of the root meristem was special in that 5–7 AT-rich domains were arranged in a strikingly regular circle on one nuclear pole (Fig. 1l, m), indicating that two or slightly more chromosomes involved in the ring can be associated with one another as a group. Moreover, some of these brightly fluorescing heterochromatic masses participating in the ring actually appeared to be made up of two paired AT-rich domains (Fig. 1l). Interestingly, pairing of the pericentromeric regions seems quite a common phenomenon in the section *Rhoeo* since from one to several “pairs” of AT-rich domains were consistently detected in the nuclei of the isolated root meristem and in the nuclei representing the 1-mm root sector (Fig. 1j, k).

### Pericentromeres associate during root development and are extensively clustered in terminally differentiated cells

No significant difference (*p* > 0.05) between collections from the isolated root meristem and those from the 1-mm root sector (Table S3) was found. Thus, root meristem cells seem to be the prevailing component of this sector. Starting from the 2nd millimeter, the association of the AT-rich domains was obviously in a steady progress, with the underlying progressive reduction of the means, median, and modal values (Fig. 2a; Table S1a; Table S3) accompanied by relevant changes in the frequency of the nuclear classes (Fig. 2b, c; Table S2a). The most spectacular was the increase in the frequency of nuclei possessing 1–2 AT-rich domains—from 1% (ring-forming variety) or ca. 3% (bivalent-forming variety) in the first millimeter to ca. 51% or 30% in the tenth millimeter. All this finally ended with ca. twice lower the mean number of AT-rich domains per nucleus in the 10-mm sector than in the root meristem (Fig. 2a; Table S1a).

In both varieties, the mean number of AT-rich domains per nucleus of the non-dividing yet metabolically highly active root hairs (Fig. 1p; Fig. 2a; Table S1a) was in the range of 2.2–2.5. Accordingly, nuclei with 1–2 or 3–4 AT-rich domains were most abundant (Fig. 2b, c; Table S2a). As in the 10-mm root sector, the frequency of nuclei with 5–6 AT-rich domains was very low (1.4–1.7%) and there was no nuclear class with 7–8 such structures (Fig. 2b, c; Table S2a).

The comparison of the two DAPI/AMD-stained non-dividing reference tissues, i.e., the leaf parenchyma and epidermis, in both varieties showed the lack of differences (*p* > 0.05, Table S3). Indeed, the two tissue types had undergone terminal differentiation characterized by extremely high 95–100% frequency of nuclei with 1–2 AT-rich domains and absence or negligible numbers of nuclei possessing 3–4 such structures (Fig. 2b, c; Table S2a). Notably, nuclei with one AT-rich domain prevailed, representing from 87% (parenchyma) to 94% (epidermis) in the ring-forming variety or from 62% (parenchyma) to 64% (epidermis) in the bivalent-forming plants (Fig. 2b, c; Table S4). Correspondingly, there was a 3.7–5.5-fold decrease of the mean number of AT-rich domains per nucleus when compared with root meristem (Fig. 2a; Table S1a).

### GC-rich genome fraction cooperates with AT-rich one for robust heterochromatic associations

**Fig. 3:**
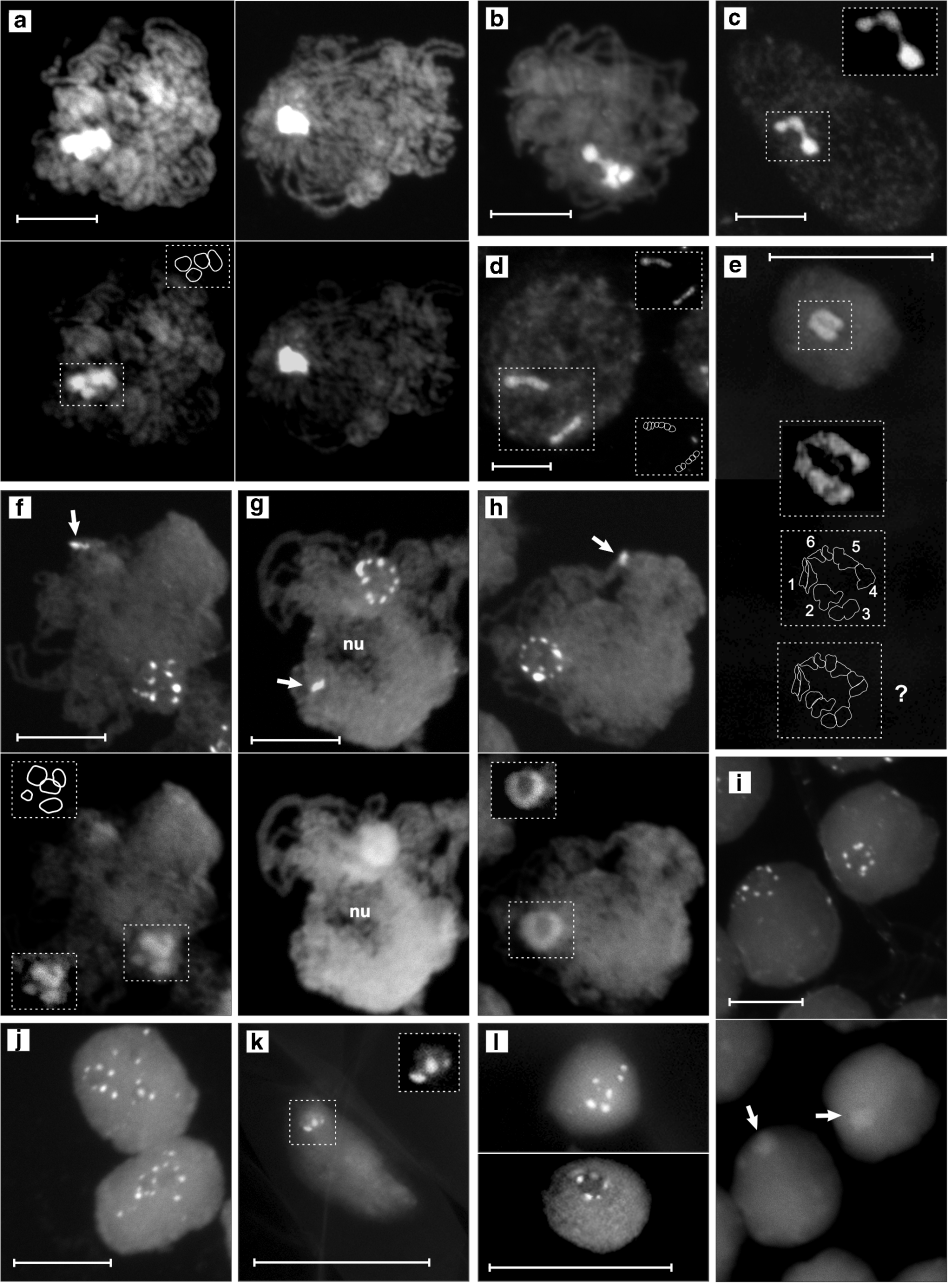
**a**–**l** AT-rich (**a**–**e**) and GC-rich (**f**–**l**) heterochromatic domains (CMA_3_/DA/DAPI technique) in cycling and differentiated nuclei of the ring-forming variety (**b**–**h**, **l**) and bivalent-forming variety (**a**, **i**–**k**) of *T. spathacea*; bars = 10 μm; nu, nucleolus; additional dash line boxes represent graphical interpretation or structural details viewed under low exposure time and alleviated contrast of the camera. **a** Pachytene nuclei viewed under normal (top panels) and low exposure (bottom panels). **b** and **c** Strongly squashed nuclei representing pachytene (**b**) and root meristem (**c**) with thin fibers connecting clustered AT-rich prericentromeric domains. **d** Root meristem nucleus with two chains consisting of side-by-side positioned pericentromeres. **e** Leaf parenchyma nucleus with a chain-like arrangement pericentromeres; the chain consists of six (1–6) domains, two of them (domain 1 and 6) consisting clearly of two side-by-side arranged subdomains which most likely represent single pericentromeres (top and middle box); such a chromatin organization can be interpreted as a ring-type collective chromocenter disturbed by squashing (bottom box). **f**–**h** Pachytene nuclei with their pericentromeric GC-rich foci (top panels) scattered (**f**) or ring-arranged (**g**, **h**); bottom panels show the same nuclei viewed in the DAPI channel; to see clearly the correlation between ring-type arrangement of the DAPI-positive heterochromatin and formation of the peripheral circle by the GC-rich pericentromeric domains, compare **f** with **h**; typically however the DAPI-rings could not be satisfactory resolved because of the high fluorescent haze of the UV illumination—see **g** as an example; arrows point to terminal GC-rich NOR sites typically fused into one spot localized opposite to the centromere pole. **i** Nuclei from the root 10th mm possessing one collective chromocenter (bottom panel, arrows) with a clear ring of CMA_3_-foci (top panel) localized peripherally around the AT-rich chromocenter core; such ring-type chromocenters were seen in those somatic nuclei of the root meristem and root sectors which possessed clearly one big AT-rich pericentromeric domain; in nuclei with more AT-rich domains, the CMA_3_-foci were scattered, as seen in **j**. **j** Root meristem nuclei with scattered CMA_3_-positive fluorescence foci. **k** and **l** CMA_3_-fluorscence of root hair (**k**), leaf parenchyma (**l** top), leaf epidermis (**l** bottom) nuclei; the ring-arrangement of the CMA_3_-foci as seen in **l** (bottom panel) was frequently observed in the three types of terminally differentiated nuclei in both varieties; however, it was not possible to state whether the foci are arranged around AT-rich heterochromatic core. Due to small size of these nuclei and a high density of their chromatin, it was not possible to distinguish their pericentromeric chromocenters in the DAPI channel when CMA_3_/DA/DAPI technique was applied

The GC-rich chromatin domains experienced a global trend for self-association similar to that of the AT-rich domains. While 4–12 or 4–17 GC-rich domains per root meristem nucleus were scored in the ring- or bivalent-forming variety, respectively, the mean was 8.2 or 9.8 (Table S1b). Correspondingly, root meristem nuclei possessing 7–10 GC-rich domains had the highest frequency (Table S2b). Since the total number of stable CMA_3_^+^ bands in the karyotype of the ring-forming variety and of the bivalent-former is 27 and 20, respectively (Golczyk et al. 2010; Golczyk 2011c), the mean numbers of CMA_3_^+^ foci per nucleus in the root meristem are then 2–3 times lower than expected for diploid cells. Surprisingly, in each variety, the mean number of GC-rich domains per pachytene nucleus was not markedly lower but even slightly higher compared with the root meristem (Fig. 2a; Table S1b), thus opposite to what could be expected based on the strong tendency for associations between meiotic AT-rich pericentromeric domains (Fig. 2a). Tight fusion of the GC-rich telomeric NORs in both varieties reflects the strong polar clustering of the terminal chromosomal sites into the meiotic telomere bouquet (Fig. 3f–h; S1b top), which mirrors the FISH-detected meiotic chromatin arrangement of the previously studied ring-forming plants (Golczyk 2011b).

The dynamics of the global self-associations of the GC-rich sites during root development closely matched the progressive attraction between the AT-rich domains (Fig. 2a). The mean number of GC-rich domains per nucleus in the 10-mm root sector and in root hairs experienced a ca. double reduction compared with the root meristem (Fig. 2a, Table S1b). Finally, it reached the lowest values ranged within 2.7–4.1 in the terminally differentiated parenchyma and epidermis (Fig. 2a, Table S1b). Thus, the global association pattern of the GC-rich genome fractions during development (Fig. 2a) indicates that both AT- and GC-rich pericentromeric domains participate in self-associations, establishing robust interchromosomal contacts.

### Internal organization of the collective chromocenter

The superior resolution and depth of focus of the EDF imaging gave the chance to see that even if the AT-rich domains were engaged in a tight and highly fluorescing association, it was frequently possible to distinguish some optically darker regions dividing a collective chromocenter into several distinct parts, especially when reducing the exposure time of the camera (Figs. 1a–c; 3a left; d–e). Yet, numerous multiple associations of the AT-rich domains were found to be too tight to resolve any internal details (Figs. 1n–p; 3a right). However, when strongly squashed, two or several higher-order domains together with thin interconnecting DAPI/AMD-positive threads could be seen, indicating the existence of intimate ectopic connections between heterologous AT-rich DNA sequences (Fig. 3b, c). The highly intriguing observations were those during which (results of applying DAPI/AMD technique) a side-by-side pericentromere juxtaposition was found in the root tip meristems of both varieties in the form of peculiar chains (Fig. 3d) or was inferred from the fluorescence pattern of a few parenchyma nuclei (Fig. 3e).

Interesting relationships were also found between AT- and GC-rich regions of a collective chromocenter. If the meiotic pericentromeric CMA_3_^+^ foci were scattered (Fig. 3f top), the AT-rich domains were also randomly arranged (Fig. 3f bottom). However, in many chromocenters whose DAPI-labeled pericentromeres tightly adhered to form a homogeneously fluorescing mass without a possibility to see any structural details (when viewed with the use of the DAPI filter set), the CMA_3_^+^ pericentromeric sites (viewed with the CMA_3_ filter set) were observed clearly to be ring-arranged on the chromocenter periphery (Fig. 3g). The ring-type peripheral disposition of the CMA_3_^+^ foci was especially clearly seen (Fig. 3h top) in those meiotic nuclei that had their AT-rich heterochromatin arranged tightly in a ring with an optically “empty” central region (Fig. 3h bottom). The peripheral ring-arrangement of the pericentromeric CMA_3_^+^ foci was observed in the pachytene nuclei of bivalent-formers (Fig. S1b) and in the root meristem and root sectors of both varieties—in large somatic nuclei which possessed clearly one big AT-rich domain (see Fig. 3i, j and explanations therein). The existence of such a chromatin arrangement in small terminally differentiated nuclei possessing one AT-rich domain could not be however ascertained because of the serious technical obstacles (see Fig. 3k, l and explanations therein).

## Discussion

### Pericentromere attraction in cycling tissues: pachytene and root tip meristem nuclei

Our results show that the ectopic self-adherence of pericentromeric regions generates a highly polarized nuclear arrangement in both cycling tissues (root meristem and meiotic cells) of each variety, with pericentromere cluster(s) localized on one nuclear pole. The very low mean number of AT-rich meiotic domains per nucleus (1.6–2.1) suggests a significance of the extensive pericentromeric associations both for bivalent- and ring-mode of meiotic division in the section *Rhoeo*, which can be related to a special genetic status of both varieties (see the last subsection of the Discussion).

Since in both varieties each pericentromere has its own single AT-rich domain, 5–6 such domains per nucleus is ca. half the diploid chromosome number (see Introduction). Thus, our data strongly suggests that chromosome pairing via pericentromeric regions as a sole or a dominant mode of association may occur in 41–48% of the root meristem nuclei (see Results). A good support for this comes from the observations on regular “pairs” formed by AT-rich domains (Fig. 1j, k).

Chromocenter pairing reported in other organisms by early cytologists was usually taken for granted as an indication of homologous pairing (Comings 1980 and literature therein). This phenomenon may be assisted by the close proximity of chromosomes that adopt a Rabl configuration with (peri)centromeric regions as pairing mediators (Hadlaczky et al. 1986; Houben et al. 1995; Martinez-Pérez et al. 1999; Prieto et al. 2004). However, in *Arabidopsis* endosperm, even without Rabl arrangement, chromosomes associate in pairs via their (peri)centromeres and other parts, possibly involving a complement from each parent (Baroux et al. 2017). In general, somatic homologous pairing is considered an exception than a rule and most organisms seem to expend considerable effort to restrict it only to some chromosomes, genomic regions, or tissues within narrow time intervals of some special developmental contexts (Westergaard 1964; Joyce et al. 2016 and literature therein). In contrast to the bivalent-forming *T. spathacea* variety, the ring-forming variety lacks pairs of homologous chromosomes in its karyotype (see Introduction). Thus, non-homologous pairwise associations or mixed-type pairings in *T. spathacea* cannot be excluded. There is a strong evidence that euchromatic and heterochromatic regions can behave differently in relation to chromosome pairing (Da Ines et al. 2014 and references therein). Likewise, non-homologous centromere pairs can be regularly formed both in meiotic and non-meiotic cells (Martinez-Pérez et al. 1999; Da Ines and White 2015 and literature therein; Corredor et al. 2007 and literature therein).

By demonstrating that nuclei with 5–6 AT-rich domains are the most frequent in the *T. spathacea* root meristem, our study locates itself in opposition to what has been revealed so far. Huskins and Steinitz (1948), who applied the Feulgen method to study root tips of the typical ring-forming variety of *T. spathacea*, found nuclei with 10–12 chromocenters to be the dominant nuclear class. Mosiołek et al. (2005) used the same heterozygous stock studied in the present work and assigned the highest ~ 72% average frequency to DAPI-stained root tip nuclei possessing 1–3 chromocenters. However, Huskins and Steinitz (Ibid.) did not find this class of nuclei at all, while 6 chromocenters per nucleus were their lowest score. By contrast, if we recalculate the frequencies of nuclear classes for both varieties (see Table S4), nuclei with 1–3 pericentromeric domains appear to be rare in our study (ca. 6%). This may result from the breakdown of the multiple pericentromere associations in the S-phase (Bartholdi 1991; Csink and Henikoff 1998). However, we have found the opposite condition deprived of pericentromeric associations to be even rarer (Table S2a). Thus, both states either are probably very short in the cell cycle or are indeed exceptional.

According to our experience, the apparently sharp conflict between previous reports (Huskins and Steinitz 1948; Mosiołek et al. 2005) and between them and the present study is most likely related to the use of non-differential techniques (Feulgen method or simple DAPI fluorescence) by the other authors (Ibid.). Such techniques stain non-specifically the whole heterochromatin fraction (e.g., both pericentromeric and telomeric heterochromatin) together with other dense chromatin lumps or irregularly shaped nuclear elements. Thus, their results can be vastly under- or overestimated, depending on the observer’s interpretation and attitude, especially when dealing with a nuclear meshwork that is in itself variable in its manifestations—see Fig. S2 for further explanations.

The regular polar (localized on one nuclear pole) circle of 5–7 heterochromatic AT-rich domains found in root meristems (Fig. 1l, m) deserves special attention. Hypothetically, such a ring-type heterochromatic structure docked in the nuclear envelope may function as a preparatory step for assembling of a common wheel-shaped prometaphase chromosomal rosette by facilitating a quick capture of kinetochores by microtubules in a ring-like manner. The centromeres of the prometaphase rosette are arranged in a ring surrounding the spindle (Magidson et al. 2011 and literature therein), and chromosome positioning within the rosette reflects interphase proximity in that it can be transmitted to daughter nuclei through metaphase-to-telophase transition (Gerlich et al. 2003; Kosak et al. 2007). Whether heterochromatic associations of *T. spathacea* formed during interphase between chromosomes of the ring render them to be juxtaposed through prophase to metaphase and/or to later stages of the cycle remain to be answered. Since 5–7 ring-arranged pericentromeric bands have been previously observed within C-banded root tip prophases of the ring-forming variety (Golczyk and Joachimiak 1999; Golczyk unpublished), a possibility of a continuum between the ring at interphase and that detected later during division is raised here.

The ring-like array of pericentromeres was found in root tip interphase and from prophase to telophase in two plants possessing Rabl-arranged cycling nuclei: *Trigonella foenum-graecum* and *Lathyrus sphaericus* (Lavania and Sharma 1980, 1984). In interphase and prophase nuclei of *L. sphaericus* (2n = 2x = 14), 5–7 ring-arranged lumps consisting of pericentromeric heterochromatin were detected with the use of C-banding, suggesting that 2–3 chromosomes involved in the ring were in both stages associated via their pericentromeric heterochromatin into one group (Lavania and Sharma 1984). The centromere ring was also described in other plants at various stages of the mitotic cycle (Mosolov 1974; Mosolov and Bondareva 1976 and literature therein; Anamthawat-Jónsson and Heslop-Harrison 1990).

### Pericentromeres associate during root development and are extensively clustered in terminally differentiated cells

Chromocenters in 67 plant species have been studied by Ceccarelli et al. (1998). A consistent observation was that the maximal number of these structures was as a rule found in the distal part of the root meristem, whereas significant heterochromatic associations, if occurred, took place at its base where the mitotic activity had ceased. It was found that the nuclear pattern produced in this region remained unchanged in differentiated tissues and a clear negative correlation between the extent of chromocenter attraction and that of RNA synthesis was reported as well (Ibid.). All this suggests that chromocenter association plays a role in or is a marker of events that have a part in the regulation of the functional activity of the nucleus and in tissue differentiation from its early stages. Indeed, heterochromatin self-associations, even if subtle, can be exploited in the process of orchestrated gene activation/repression during development/differentiation, since they create chromosomal order (nuclear constraints) and drive separation of the silencing heterochromatic compartment from the active euchromatic one (Wijchers et al. 2015; Ostromyshenskii et al. 2018 and literature therein).

However, fixation of the heterochromatin associations as soon as the mitotic division activity declines obviously does not fit to what was observed by us in both varieties (see Fig. 2a). We showed that the association process increased during differentiation and development, ending up with one chromocenter in terminally differentiated tissues. Thus, the observed increase in the level of pericentromere association is unlikely to be a merely passive consequence of centromere disposition at the poles of the mitotic spindle during the late anaphase/telophase of the last division. Clustering and relocation of pericentromeric regions in differentiated cells have been detected in animals/humans, suggesting that the changes are an active process with functional significance (Ostromyshenskii et al. 2018 and literature therein). It is now being increasingly debated that the association of the centromeric regions or heterochromatic pericentromeres can be actively regulated via their connection with the nuclear envelope environment (Padeken and Heun 2014; Poulet et al. 2017 and literature therein).

In contrast to our findings, Huskins and Steinitz (1948) reported that the number of the chromocenters within 1–5 mm of the root in the typical heterozygous variety of *T. spathacea* increased on average twice as the root tissues grew and differentiated, i.e., from ~ 11 in the 1st millimeter of the root where meristematic cells prevailed to ~ 21 in the fully differentiated 5th millimeter. Although the conflict between their data and that obtained by us is tremendous, it can be explainable by the usage of non-differential techniques (Feulgen method or simple DAPI fluorescence) by the previous authors (see previous subsection and Fig. S2).

Our study locates *T. spathacea* in a highly interesting position among plants, i.e., as an organism which clearly acquired a high competence for chromocenter association while maintaining the non-random highly polarized nuclear architecture. The data reported by Ceccarelli et al. (1998) showed that frequently the highest degree of chromocenter associations is expressed by the leaf mesophyll, with 5.3 as the lowest reported mean number of chromocenters per nucleus. However, it is still ca. 3.7–4.6 times more than in the case of the *T. spathacea* varieties studied here. From 67% (ring-forming variety) to 78% (bivalent-forming variety), root meristem nuclei had the number of the DAPI\AMD foci equal to or lower than half the pericentromere number in the karyotype (Table S2a). In the 10-mm root section and in the studied differentiated tissues, the frequency of this nuclear class was 100% (Table S2a). As a comparison, in meristems and differentiated tissues of *Arabidopsis thaliana* (*Brassicaceae*, 2n = 2x = 10), the frequency of nuclei with this number of FISH-labeled pericentromeric foci, as inferred from the supplemental data provided by Berr and Schubert (2007), is zero or negligible. In general, pericentromeres in *Arabidopsis* express rather weak attraction during differentiation: it does not go at all or too far beyond the average association level found in a meristematic tissue (Ibid.). This species is a widely used model for elucidation of rules governing the non-random nuclear chromatin arrangement in plants; yet, its pericentromeres are typically scattered throughout nuclear periphery without any apparent order (Schubert et al. 2014 and literature therein).

### Heterochromatin self-attraction and structure of collective chromocenters

The mechanism of heterochromatin self-attraction or stickiness is likely based on the similarity of the structure and biochemical properties of chromatin sites that connect with each other (Barr and Ellison 1972; Cerda et al. 1999; Pedrosa et al. 2001). In general, both DNA-DNA interactions and contacts via chromatin-associated proteins may be involved and determine the degree of association intimacy (Barr and Ellison 1972; Mayfield and Ellison 1975; Cerda et al. 1999; Sage and Csink 2003; Belyaeva et al. 2006; Smith and Weiler 2010; Jagannathan et al. 2019). The calculated mean numbers of AT- and GC-rich chromatin domains per nucleus together with frequencies of the distinguished nuclear classes (Fig. 2a–c) strongly argue that in the section *Rhoeo*, AT-rich genome fractions cooperate with GC-rich ones for heterochromatic associations, that is, both heterochromatin parts have the ability to self-associate and exploit this ability to modify the nuclear architecture. While fusion was the association type actually experienced by GC-rich chromatin clusters on the cytological level, it is rather impossible to support explicitly wholesale intimate linking as being a sole interaction mode between the AT-rich domains. The results indicate that the heterochromatin of a collective chromocenter (both in meiotic and somatic cells) comprises separate higher-order domains consisting of one to several pericentromeres (Fig. 1b) and that these domains are connected via chromatin fibers (Fig. 3b, c). Such a modular architecture is likely a consequence of the stepwise pericentromere association process, which starts from lower-order associations (pairs, triples) to finally yield complicated multiple aggregates with one collective chromocenter in terminally differentiated tissues as the ultimate nuclear configuration.

**Fig. 4:**
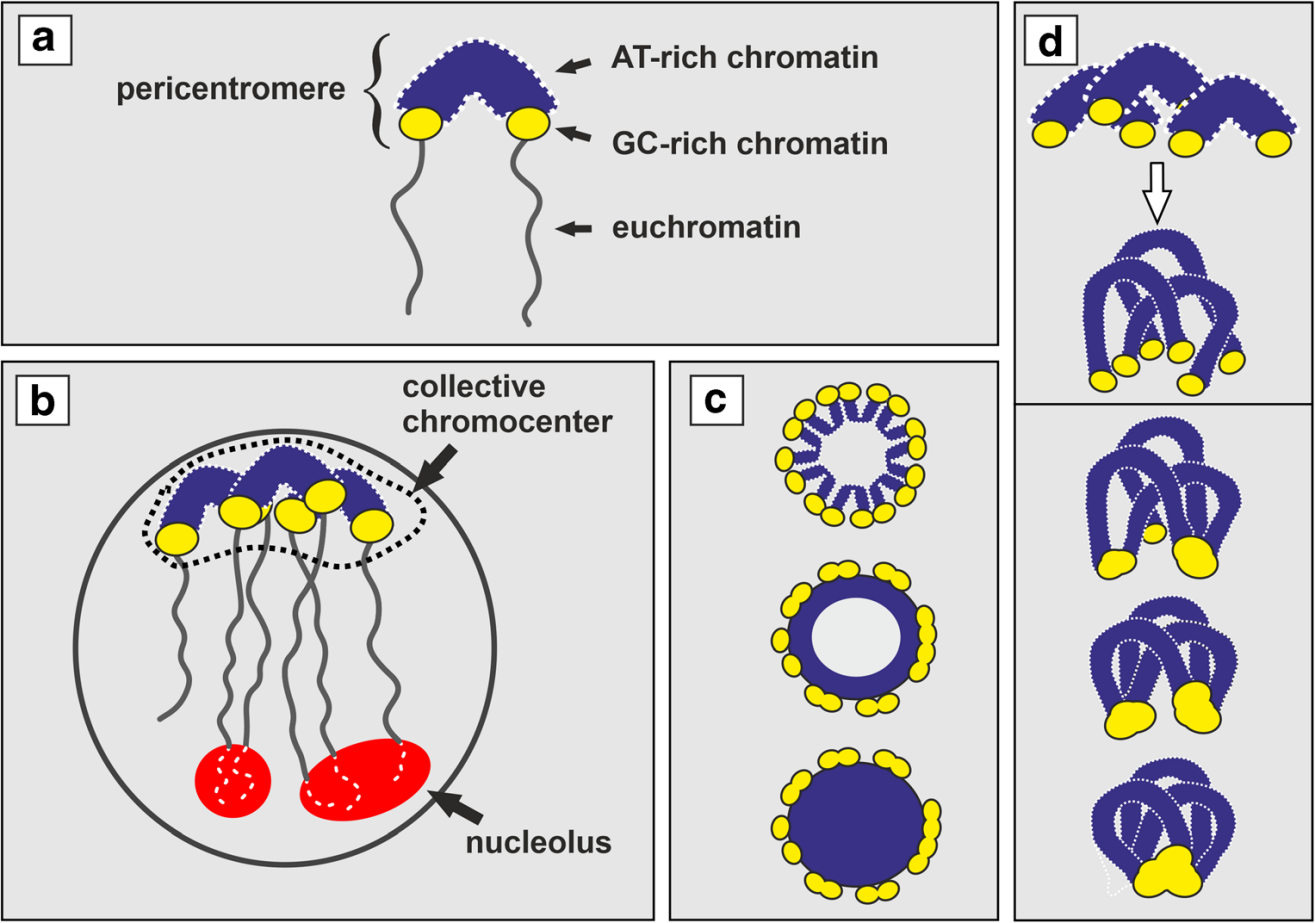
**a**–**d** Multiple nuclear constraints operate in *Tradescantia* section *Rhoeo*. **a** Pericentromere structure. **b** A general universal Rabl nuclear organization with heterochromatic pericentromeres clustered on one pole to form collective chromocenter(s) and differently fused terminal NORs forming joint nucleolus or nucleoli. **c** Planar view of a ring-type pericentromeric collective chromocenter; when GC-rich pericentromeric domains are seen peripherally around the AT-rich chromocenter core, a ring of side-by-side positioned pericentromeres formed within the Rabl organization is the only possibly explanation (top); ideally such an arrangement can be viewed as the AT-rich heterochromatic ring in the DAPI channel (middle); frequently however such rings could not be satisfactorily resolved because of the high fluorescent haze of the UV illumination (bottom); GC-rich domains can be subjected to different degrees of fusion reducing the number of the corresponding fluorescent spots. **d** Pericentromere elongation during interphase (top panel) increases the fusigenic potential of the GC-rich domains by allowing their multiple contacts (bottom panel)

A circle of the meiotic pericentromeric CMA_3_^+^ foci arranged peripherally in relation to the ring-shaped AT-rich chromocenter core (Fig. 3g, h) reflects a highly stringent side-by-side positioning of the pericentromeres involved (see Fig. 4a–c for explanations). It also suggests the high potential of a meiotic collective chromocenter for restructuring from the modular architecture into the ring-like side-by-side pericentromere arrangement (compare Fig. 3f with 3 h). In agreement with our findings, the C-banded continuous pachytene ring with empty space inside was previously depicted in the ring-forming plants (Golczyk 2011b). However, we show that the ring-type chromocenter with a clear circle of CMA_3_-foci localized peripherally around the AT-rich core cannot be exclusively linked to meiotic catenation, but should rather be viewed as a more universal temporary product of mitotic and meiotic chromatin dynamics of the section *Rhoeo* (Fig. S1, Fig. 3i). It remains to be answered whether the chain-like chromocenters with side-by-side juxtaposed pericentromeres detected with the use of the DAPI/AMD technique (Fig. 3d, e) in somatic cells reflect the failure to assemble the ring-type collective chromocenter or result from disruption of the latter due to squashing, or are caused by other mechanisms.

As far as we are aware, the ring or chain of side-by-side juxtaposed (peri)centromeres in pachytene or somatic interphase has not been found so far in any other plant species. However, rings or lines consisting of prekinetochores have been detected during mammalian interphase (Bartholdi 1991; Zalensky et al. 1993; He and Brinkley 1996). A chromocenter consisting of a regular compact pericentromere ring with the spindle pole body at its hub is actively maintained throughout interphase of the budding yeast (Loidl 2003 and literature therein).

The establishment of intrachromocentric contacts between GC-rich pericentromeric domains seems a flexible process yielding the most extreme multiple fusions in differentiated cells (Fig. 2a; Table S1b). This may be facilitated by pericentromere elongation due to the ability of heterochromatin to disperse/decondense (Schubert et al. 2014 and literature therein)—see Fig. 4a, d for explanation. However, such a structural plasticity should be restricted if the pericentromeres of a collective chromocenter were more condensed and/or transiently arranged in a ring. The most stringent version of the ring-type intrachromocentral organization occurs in meiotic prophase, i.e., when heavily condensed pericentromeres adhere side-by-side during pachytene (Fig. 4a–c). This may at least partly explain why the global level of the associations between pachytene GC-rich domains in both varieties is not as high as expected.

### Multiple nuclear constraints operate in the section *Rhoeo*

All our results indicate that unusual spectrum of multiple nuclear constraints involving heterochromatic genome fraction is shared by meiotic and somatic nuclei of the ring- and bivalent-forming variety. These constraints are strong nuclear polarization and robust multiple AT- and GC-rich heterochromatic associations, including the ring or chain of side-by-side juxtaposed (peri)centromeres.

The intensity of the heterochromatic associations may be brought about by specific phyletic traits acquired by a species or a higher-level taxon (Ceccarelli et al. 1998). The non-recombining and strongly reshuffled genomes of permanently heterozygous species likely need a high degree of nuclear order and compartmentation (Golczyk et al. 2014). The nuclear constraints may have primarily evolved to help in regular pairing and disjunction, thus preventing gross genetic imbalances (discussed in Golczyk 2013). The karyotypic changes that caused recombination to cease during evolution of permanent translocation heterozygosity may have left sites of multiple homology or other serious structural complications on chromosomes of not one but of both genomes, as in the case of *Oenothera* (Cleland 1972). In such a view, the remarkable potential for nuclear constraints is likely to be of functional importance also for the bivalent-forming variety (which possess one of the translocation genomes in double dose). Accordingly, in the *Oenothera* translocation system, pericentromeric regions also remain clustered and generate Rabl-polarization in cells of the root tip meristem and throughout pachytene both in ring-forming plants and in regular bivalent-formers (Golczyk et al. 2008, 2014). The latter include *Oe. glazioviana* strain blandina de Vries—a representative of homozygous lines that can segregate on different occasions from permanent heterozygotes (Cleland 1972). This *Oenothera* strain shares the whole set of chromosomal structural details together with chromatin dynamics and its epigenetic signatures with the permanently heterozygous species (Golczyk et al. 2014). Interestingly, extensive AFLP-genotyping provides evidence that meiotic crossing-over in the bivalent-forming *Oenothera* species is drastically reduced and confined exclusively to the very chromosome ends (Rauwolf et al. 2011), as in the ring-forming plants (Rauwolf et al. 2008), suggesting a special genetic status of the homozygous individuals. Thus, the genetic condition of the studied here bivalent-forming variety of *T. spathacea* may be similar to that of blandina de Vries (see Golczyk 2013 and literature therein). Nuclear constraints may potentially promote homology search and pairing in face of recombination dysfunctions (Barzel and Kupiec 2008). For example, the ring-type collective chromocenter could be hypothetically a transient step of the recombination-independent sorting process that helps in the establishment of recombination-dependent homologous pairing (Da Ines and White 2015 and literature therein).

## Conclusions

This report is the first comprehensive evidence that the unusually extensive association of heterochromatic pericentromeres in both ring- and bivalent-forming *T. spathacea* involves developmentally regulated active self-attraction of their AT- and GC-rich domains and increases with tissue differentiation. The striking nuclear polarization and robust heterochromatic associations are the widespread higher-order nuclear constraints shared between cycling and differentiated cells as well as between somatic and meiotic tissues of the section *Rhoeo*. However, another somatic/meiotic constraint is the internal organization of the pericentromeric collective chromocenter, i.e., the arrangement of its AT- and GC-rich components. The unusual collection of the shared non-random chromatin patterns, e.g., pericentromere pairing and their clustering into 5–7 ring-arranged chromocenters in root tip interphase, the ring or chain of side-by-side juxtaposed (peri)centromeres in pachytene or somatic interphase, deserves special attention. Unique for plants is that the collective pericentromeric chromocenter does not seem to be an accidental structure since formed with involvement of definite sites and according to a certain order (Fig. 4). Thus, in contrast to model plants with sequenced genomes, such as *Arabidopsis*, *T. spathacea* is a favorable model for studies of non-random nuclear architecture, including chromocenter association and structure, and their biological significance.

## Electronic supplementary material

Fig. S1 Pachytene nuclei of the bivalent-forming variety of T.spathacea. a, DAPI/AMD technique; nuclei with 1–4 chromocenters formed by AT-rich pericentromeric domains. b, CMA3/DA/DAPI technique; DAPI fluorescence in the bottom panel; a ring of CMA3-foci (top panel) is localized peripherally in relation to the AT-rich chromocenter core (bottom panel, arrowhead); arrow (top panel) indicates terminal GC-rich NORs fused into one cluster. Bars = 10 μm (PNG 753 kb).

High resolution image (TIF 2746 kb).

Fig S2 A typical result of the non-differential simple DAPI staining. Nucleus from isolated root meristem of the ring-forming variety. AT-rich pericentromeric heterochromatin does not stand out clearly from other optically dense regions. The latter have visibly granular or sometimes thread-like appearance. Here, a decision to interpret many of chromatin lumps as pericentromeric heterochromatin will depend on personal experience and research intuition, but unfortunately also on imagination. With the “cautious” interpretation, there is a tendency to select the biggest lumps and to underestimate the number of pericentromeric heterochromatin domains per nucleus, while the opposite approach may lead to an excessive increase of this number. The same interpretation problems are met when Feulgen method is applied (data not shown, Golczyk unpbl.). As a result of differential DAPI/AMD technique, the non-specific fluorescence of the other denser nuclear regions is quenched and form a rather uniform background of low emission for the bright and now highly contrasting fluorescence of the AT-rich pericentromeric domains (see: Fig. 1). Bar = 10 μm (PNG 252 kb).

High resolution image (TIF 828 kb).

Table S1 a–b AT-rich (a) and GC-rich (b) domains, basic parameters. (I) = ring-forming variety; (II) = bivalent-forming variety. MND = mean number of domains per nucleus; sd = standard deviation; Med., Mod. = median and modal values; Min., Max. = minimal and maximal number of domains per nucleus; 25/75 = 25th and 75th percentile; 5/95 = 5th and 95th percentile (DOCX 19 kb).

Table S2 a-b AT-rich (a) and GC-rich (b) domains – frequency (%) of nuclear classes in ring-forming variety (I) and bivalent-forming variety (II). 1–2, 3–4, 5–6, etc. = nuclear classes characterized by the presence of 1–2, 3–4, 5–6, etc. domains per nucleus. Standard deviations in the brackets (DOCX 24 kb).

Table S3 The results of statistical analysis. Out of all the possible pairwise intra-varietal comparisons only 14 [■] showed no significant difference (*p* > 0.05). The rest of listed comparisons and all the remaining comparisons not listed here disclosed significant difference (*p* < 0.05) * * As seen in the Table S3, statistically identical were only some adjacent root sectors, which points to a gradual character of the changes in the interphase architecture as the root grows. In line, significant differences appeared as soon as the alternate root sectors were compared. Thus, the used statistics is compatible with the steady reduction of the number of AT- or GC-rich chromatin domains per nucleus during root development (DOC 50 kb).

Table S4 AT-rich domains in ring-forming variety (I) and bivalent-forming variety (II). Frequency (%) of nuclear classes with 1–3 AT-rich domains (DOCX 15 kb).

## Abbreviations

AMD: Actinomycin D
CMA_3_: Chromomycin A_3_
DA: Distamycin A
DAPI: 4′,6-diamidino-2-phenylindole
