# Supplementary figures and images for: Pericentromere clustering in Tradescantia section Rhoeo involves self-associations of AT- and GC-rich heterochromatin fractions, is developmentally regulated, and increases during differentiation
Source: Chromosoma. 2020 Jul 17;129(3):227–42. doi: 10.1007/s00412-020-00740-x (PMC7666280; doi:10.1007/s00412-020-00740-x)

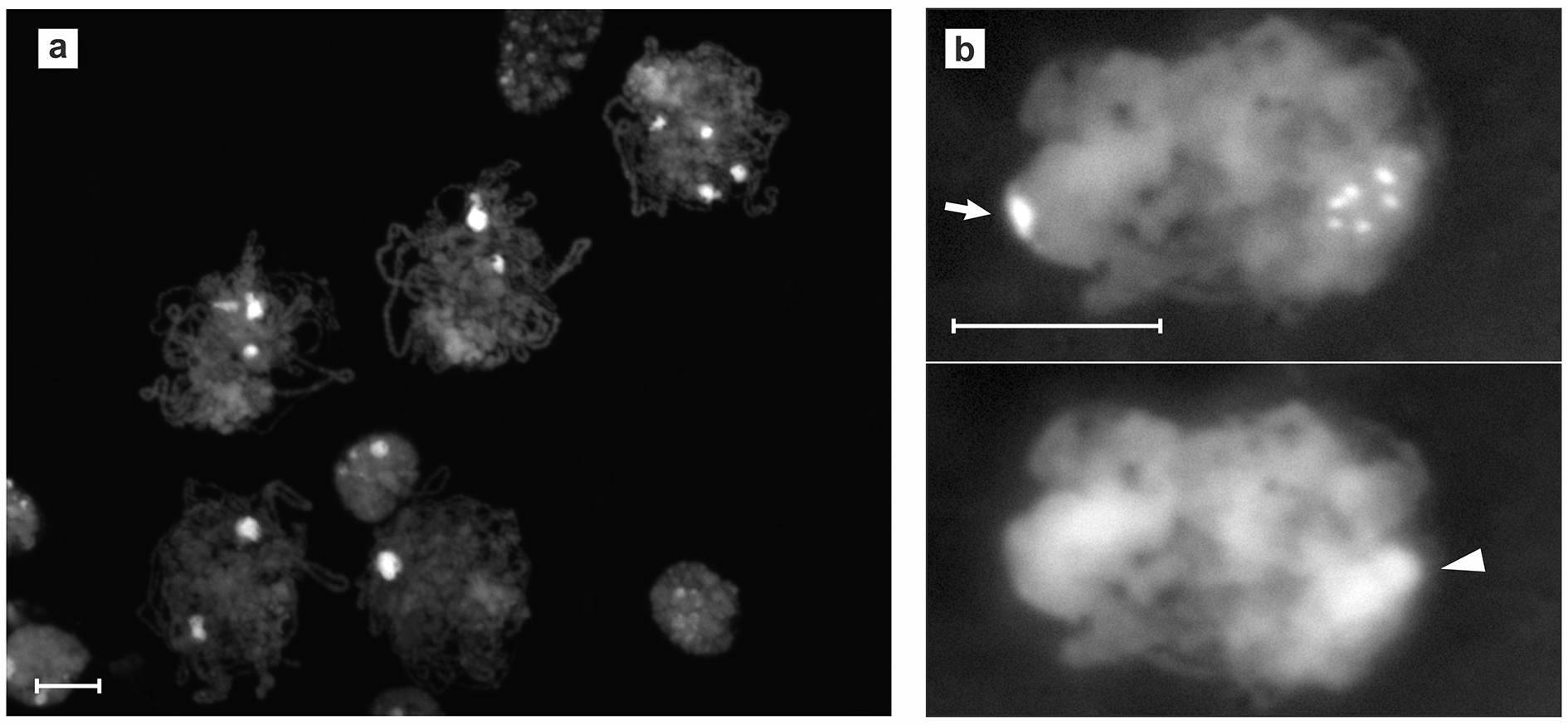

Supplement: Supplementary file 1 — Pachytene nuclei of the bivalent-forming variety of T.spathacea. a, DAPI/AMD technique; nuclei with 1–4 chromocenters formed by AT-rich pericentromeric domains. b, CMA3/DA/DAPI technique; DAPI fluorescence in the bottom panel; a ring of CMA3-foci (top panel) is localized peripherally in relation to the AT-rich chromocenter core (bottom panel, arrowhead); arrow (top panel) indicates terminal GC-rich NORs fused into one cluster. Bars = 10 μm (PNG 753 kb). [file 412_2020_740_Fig5_ESM.png]

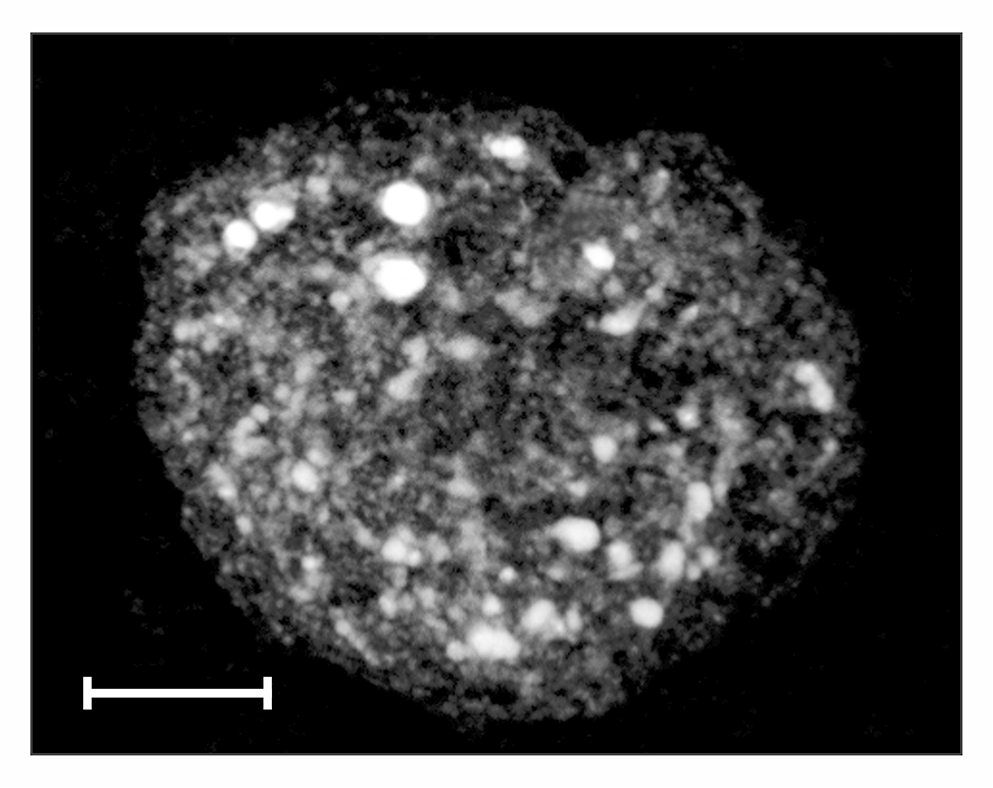

Supplement: Supplementary file 3 — A typical result of the non-differential simple DAPI staining. Nucleus from isolated root meristem of the ring-forming variety. AT-rich pericentromeric heterochromatin does not stand out clearly from other optically dense regions. The latter have visibly granular or sometimes thread-like appearance. Here, a decision to interpret many of chromatin lumps as pericentromeric heterochromatin will depend on personal experience and research intuition, but unfortunately also on imagination. With the “cautious” interpretation, there is a tendency to select the biggest lumps and to underestimate the number of pericentromeric heterochromatin domains per nucleus, while the opposite approach may lead to an excessive increase of this number. The same interpretation problems are met when Feulgen method is applied (data not shown, Golczyk unpbl.). As a result of differential DAPI/AMD technique, the non-specific fluorescence of the other denser nuclear regions is quenched and form a rather uniform background of low emission for the bright and now highly contrasting fluorescence of the AT-rich pericentromeric domains (see: Fig. 1). Bar = 10 μm (PNG 252 kb). [file 412_2020_740_Fig6_ESM.png]

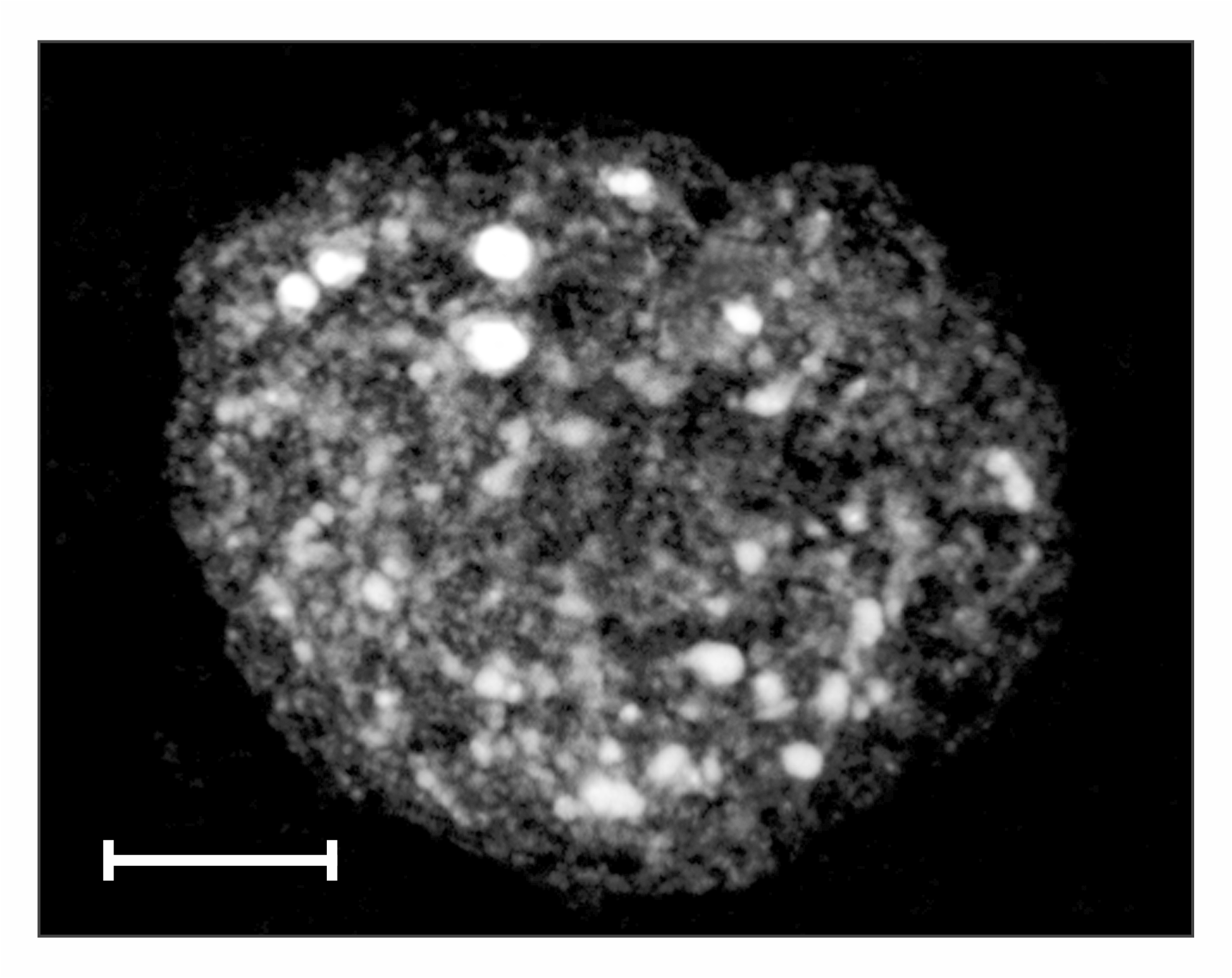

Supplement: Supplementary file 4 — High resolution image (TIF 828 kb). [file 412_2020_740_MOESM2_ESM.tif]
