# Supplementary material for: Pericentromere clustering in Tradescantia section Rhoeo involves self-associations of AT- and GC-rich heterochromatin fractions, is developmentally regulated, and increases during differentiation
Source: Chromosoma. 2020 Jul 17;129(3):227–42. doi: 10.1007/s00412-020-00740-x (PMC7666280; doi:10.1007/s00412-020-00740-x)
Supplement: Supplementary file 5 — a–b AT-rich (a) and GC-rich (b) domains, basic parameters. (I) = ring-forming variety; (II) = bivalent-forming variety. MND = mean number of domains per nucleus; sd = standard deviation; Med., Mod. = median and modal values; Min., Max. = minimal and maximal number of domains per nucleus; 25/75 = 25th and 75th percentile; 5/95 = 5th and 95th percentile (DOCX 19 kb). [file 412_2020_740_MOESM3_ESM.docx]

|  |  | |  | |  | |  | |  | |  | |  | |  | |  | |  | |  | |
| --- | --- | --- | --- | --- | --- | --- | --- | --- | --- | --- | --- | --- | --- | --- | --- | --- | --- | --- | --- | --- | --- | --- |
|  | **MP** | | **RM** | | **1 mm** | | **2 mm** | | **3 mm** | | **4 mm** | | **5 mm** | | **10 mm** | | **RH** | | **LP** | | **LE** | |
|  |  | |  | |  | |  | |  | |  | |  | |  | |  | |  | |  | |
|  | **I** | **II** | **I** | **II** | **I** | **II** | **I** | **II** | **I** | **II** | **I** | **II** | **I** | **II** | **I** | **II** | **I** | **II** | **I** | **II** | **I** | **II** |
|  |  |  |  |  |  |  |  |  |  |  |  |  |  |  |  |  |  |  |  |  |  |  |
|  |  |  |  |  |  |  |  |  |  |  |  |  |  |  |  |  |  |  |  |  |  |  |
| **MND** | **1,61** | **2,11** | **5,87** | **5,20** | **6,17** | **4,91** | **5,14** | **4,27** | **4,20** | **3,79** | **3,90** | **3,45** | **3,69** | **3,25** | **2,70** | **3,03** | **2,22** | **2,47** | **1,14** | **1,42** | **1,06** | **1,40** |
| **sd** | **0,81** | **0,92** | **1,58** | **1,75** | **1,87** | **1,44** | **1,43** | **1,33** | **1,15** | **1,25** | **1,17** | **1,09** | **1,22** | **1,07** | **1,08** | **0,98** | **0,94** | **0,90** | **0,36** | **0,56** | **0,24** | **0,57** |
|  |  |  |  |  |  |  |  |  |  |  |  |  |  |  |  |  |  |  |  |  |  |  |
|  |  |  |  |  |  |  |  |  |  |  |  |  |  |  |  |  |  |  |  |  |  |  |
| **Med.** | **1** | **2** | **6** | **5** | **6** | **5** | **5** | **4** | **4** | **4** | **4** | **3** | **4** | **3** | **2** | **3** | **2** | **2** | **1** | **1** | **1** | **1** |
|  |  |  |  |  |  |  |  |  |  |  |  |  |  |  |  |  |  |  |  |  |  |  |
| **Mod.** | **1** | **2** | **6** | **5** | **6** | **5** | **5** | **4** | **4** | **3** | **4** | **4** | **3** | **3** | **2** | **3** | **2** | **2** | **1** | **1** | **1** | **1** |
|  |  |  |  |  |  |  |  |  |  |  |  |  |  |  |  |  |  |  |  |  |  |  |
| **Min.** | **1** | **1** | **1** | **1** | **2** | **2** | **2** | **1** | **2** | **1** | **1** | **1** | **1** | **1** | **1** | **1** | **1** | **1** | **1** | **1** | **1** | **1** |
|  |  |  |  |  |  |  |  |  |  |  |  |  |  |  |  |  |  |  |  |  |  |  |
| **Max.** | **5** | **5** | **11** | **12** | **12** | **10** | **10** | **9** | **8** | **10** | **8** | **7** | **8** | **7** | **6** | **6** | **5** | **5** | **3** | **3** | **2** | **3** |
|  |  |  |  |  |  |  |  |  |  |  |  |  |  |  |  |  |  |  |  |  |  |  |
| **25/75** | **1/2** | **1/3** | **5/7** | **4/6** | **5/7** | **4/6** | **4/6** | **3/5** | **3/5** | **3/5** | **3/5** | **3/4** | **3/4** | **3/4** | **2/3** | **2/4** | **2/3** | **2/3** | **1/1** | **1/2** | **1/1** | **1/2** |
| **/** |  |  |  |  |  |  |  |  |  |  |  |  |  |  |  |  |  |  |  |  |  |  |
| **5/95** | **1/3** | **1/4** | **3/9** | **3/8** | **3/9** | **3/7** | **3/8** | **2/6** | **2/6** | **2/6** | **2/6** | **2/5** | **2/6** | **2/5** | **1/5** | **2/5** | **1/4** | **1/4** | **1/2** | **1/2** | **1/2** | **1/2** |
|  |  |  |  |  |  |  |  |  |  |  |  |  |  |  |  |  |  |  |  |  |  |  |

**Table S1a**

**Table S1b**

|  |  | |  | |  | |  | |  | |  | |  | |  | |  | |  | |  | |
| --- | --- | --- | --- | --- | --- | --- | --- | --- | --- | --- | --- | --- | --- | --- | --- | --- | --- | --- | --- | --- | --- | --- |
|  | **MP** | | **RM** | | **1 mm** | | **2 mm** | | **3 mm** | | **4 mm** | | **5 mm** | | **10 mm** | | **RH** | | **LP** | | **LE** | |
|  |  | |  | |  | |  | |  | |  | |  | |  | |  | |  | |  | |
|  | **I** | **II** | **I** | **II** | **I** | **II** | **I** | **II** | **I** | **II** | **I** | **II** | **I** | **II** | **I** | **II** | **I** | **II** | **I** | **II** | **I** | **II** |
|  |  |  |  |  |  |  |  |  |  |  |  |  |  |  |  |  |  |  |  |  |  |  |
|  |  |  |  |  |  |  |  |  |  |  |  |  |  |  |  |  |  |  |  |  |  |  |
| **MND** | **9,48** | **10,72** | **8,21** | **9,84** | **8,19** | **9,33** | **7,94** | **8,71** | **6,90** | **6,88** | **5,45** | **6,29** | **5,46** | **6,08** | **4,28** | **5,40** | **3,94** | **4,34** | **2,66** | **3,85** | **3,40** | **4,07** |
| **sd** | **1,98** | **2,65** | **1,54** | **2,43** | **2,07** | **2,95** | **1,70** | **2,65** | **1,80** | **2,36** | **1,52** | **1,94** | **1,56** | **1,93** | **1,48** | **2,05** | **0,99** | **1,36** | **0,91** | **1,20** | **0,82** | **1,60** |
|  |  |  |  |  |  |  |  |  |  |  |  |  |  |  |  |  |  |  |  |  |  |  |
|  |  |  |  |  |  |  |  |  |  |  |  |  |  |  |  |  |  |  |  |  |  |  |
| **Med.** | **9** | **10** | **8** | **10** | **8** | **9** | **8** | **9** | **7** | **7** | **5** | **6** | **5** | **6** | **4** | **5** | **4** | **4** | **3** | **4** | **3** | **4** |
|  |  |  |  |  |  |  |  |  |  |  |  |  |  |  |  |  |  |  |  |  |  |  |
| **Mod.** | **9** | **10** | **8** | **8** | **8** | **8** | **7** | **9** | **7** | **8** | **4** | **5** | **4** | **5** | **3** | **5** | **4** | **4** | **2** | **3** | **4** | **5** |
|  |  |  |  |  |  |  |  |  |  |  |  |  |  |  |  |  |  |  |  |  |  |  |
| **Min.** | **5** | **5** | **4** | **4** | **3** | **2** | **4** | **3** | **3** | **2** | **3** | **2** | **2** | **2** | **1** | **1** | **1** | **2** | **1** | **1** | **1** | **1** |
|  |  |  |  |  |  |  |  |  |  |  |  |  |  |  |  |  |  |  |  |  |  |  |
| **Max.** | **17** | **18** | **12** | **17** | **14** | **18** | **12** | **17** | **11** | **16** | **11** | **13** | **10** | **14** | **9** | **14** | **7** | **10** | **5** | **9** | **5** | **9** |
|  |  |  |  |  |  |  |  |  |  |  |  |  |  |  |  |  |  |  |  |  |  |  |
| **25/75** | **8/10** | **9/12** | **7/9** | **8/11** | **7/10** | **8/11** | **7/9** | **7/10** | **6/8** | **5/8** | **4/6** | **5/8** | **4/7** | **5/7** | **3/5** | **4/7** | **3/5** | **3/5** | **2/3** | **3/5** | **3/4** | **3/5** |
|  |  |  |  |  |  |  |  |  |  |  |  |  |  |  |  |  |  |  |  |  |  |  |
| **5/95** | **6/13** | **7/16** | **6/11** | **6/14** | **5/12** | **5/15** | **5/11** | **5/13** | **4/10** | **3/11** | **3/8** | **3/9** | **3/8** | **3/9** | **2/7** | **2/9** | **2/5** | **3/7** | **1/4** | **2/6** | **2/5** | **2/7** |
|  |  |  |  |  |  |  |  |  |  |  |  |  |  |  |  |  |  |  |  |  |  |  |
