# Supplementary material for: Pericentromere clustering in Tradescantia section Rhoeo involves self-associations of AT- and GC-rich heterochromatin fractions, is developmentally regulated, and increases during differentiation
Source: Chromosoma. 2020 Jul 17;129(3):227–42. doi: 10.1007/s00412-020-00740-x (PMC7666280; doi:10.1007/s00412-020-00740-x)
Supplement: Supplementary file 6 — a-b AT-rich (a) and GC-rich (b) domains – frequency (%) of nuclear classes in ring-forming variety (I) and bivalent-forming variety (II). 1–2, 3–4, 5–6, etc. = nuclear classes characterized by the presence of 1–2, 3–4, 5–6, etc. domains per nucleus. Standard deviations in the brackets (DOCX 24 kb). [file 412_2020_740_MOESM4_ESM.docx]

|  |  | |  | |  | |  | |  | |  | |  | |  | |  | |  | |  | |
| --- | --- | --- | --- | --- | --- | --- | --- | --- | --- | --- | --- | --- | --- | --- | --- | --- | --- | --- | --- | --- | --- | --- |
|  | **MP** | | **RM** | | **1 mm** | | **2 mm** | | **3 mm** | | **4 mm** | | **5 mm** | | **10 mm** | | **RH** | | **LP** | | **LE** | |
|  |  | |  | |  | |  | |  | |  | |  | |  | |  | |  | |  | |
|  | **I** | **II** | **I** | **II** | **I** | **II** | **I** | **II** | **I** | **II** | **I** | **II** | **I** | **II** | **I** | **II** | **I** | **II** | **I** | **II** | **I** | **II** |
|  |  |  |  |  |  |  |  |  |  |  |  |  |  |  |  |  |  |  |  |  |  |  |
|  |  |  |  |  |  |  |  |  |  |  |  |  |  |  |  |  |  |  |  |  |  |  |
| **1 - 2** | **86,9** | **69,2** | **1,4** | **3,1** | **1,0** | **2,7** | **1,6** | **7,5** | **6,2** | **10,9** | **10,7** | **20,8** | **15,3** | **24,2** | **50,8** | **30,1** | **62,6** | **51,5** | **99,4** | **95,8** | **100,0** | **95,3** |
|  | **(4,3)** | **(9,9)** | **(1,3)** | **(2,6)** | **(1,2)** | **(2,0)** | **(0,4)** | **(7,2)** | **(2,9)** | **(4,3)** | **(6,1)** | **(7,9)** | **(8,1)** | **(7,7)** | **(8,0)** | **(1,4)** | **(4,9)** | **(7,6)** | **(0,8)** | **(2,4)** | **(0,0)** | **(3,2)** |
|  |  |  |  |  |  |  |  |  |  |  |  |  |  |  |  |  |  |  |  |  |  |  |
| **3 - 4** | **12,7** | **30,4** | **17,9** | **34,6** | **18,1** | **37,0** | **33,1** | **50,0** | **54,7** | **59,5** | **61,5** | **63,2** | **62,5** | **63,7** | **43,5** | **62,8** | **36,0** | **46,8** | **0,6** | **4,2** | **–** | **4,7** |
|  | **(4,0)** | **(9,4)** | **(2,8)** | **(19,2)** | **(2,4)** | **(16,4)** | **(3,4)** | **(16,8)** | **(9,9)** | **(7,2)** | **(5,9)** | **(5,4)** | **(7,1)** | **(5,9)** | **(7,8)** | **(2,7)** | **(5,3)** | **(7,2)** | **(0,8)** | **(2,4)** | **–** | **(3,2)** |
|  |  |  |  |  |  |  |  |  |  |  |  |  |  |  |  |  |  |  |  |  |  |  |
| **5 - 6** | **0,4** | **0,4** | **47,6** | **40,7** | **40,9** | **46,7** | **49,0** | **37,5** | **36,6** | **26,7** | **26,1** | **15,3** | **20,1** | **11,9** | **5,7** | **7,1** | **1,4** | **1,7** | **–** | **–** | **–** | **–** |
|  | **(0,4)** | **(0,7)** | **(6,0)** | **(8,4)** | **(3,8)** | **(8,5)** | **(4,3)** | **(21,1)** | **(9,3)** | **(6,9)** | **(8,9)** | **(7,8)** | **(7,1)** | **(8,6)** | **(1,3)** | **(3,5)** | **(0,4)** | **(0,9)** | **–** | **–** | **–** | **–** |
|  |  |  |  |  |  |  |  |  |  |  |  |  |  |  |  |  |  |  |  |  |  |  |
| **7 - 8** | **–** | **–** | **28** | **17,5** | **30,5** | **12,2** | **14,5** | **4,5** | **2,5** | **2,5** | **1,7** | **0,7** | **2,1** | **0,2** | **–** | **–** | **–** | **–** | **–** | **–** | **–** | **–** |
|  | **–** | **–** | **(3,2)** | **(14,2)** | **(5,6)** | **(8,9)** | **(6,0)** | **(4,2)** | **(2,1)** | **(3,2)** | **(0,7)** | **(0,8)** | **(2,9)** | **(0,4)** | **–** | **–** | **–** | **–** | **–** | **–** | **–** | **–** |
|  |  |  |  |  |  |  |  |  |  |  |  |  |  |  |  |  |  |  |  |  |  |  |
| **9 - 10** | **–** | **–** | **4,8** | **3,4** | **7,9** | **1,4** | **1,8** | **0,5** | **–** | **0,4** | **–** | **–** | **–** | **–** | **–** | **–** | **–** | **–** | **–** | **–** | **–** | **–** |
|  | **–** | **–** | **(2,7)** | **(3,6)** | **(2,7)** | **(1,5)** | **(1,9)** | **(0,6)** | **–** | **(0,9)** | **–** | **–** | **–** | **–** | **–** | **–** | **–** | **–** | **–** | **–** | **–** | **–** |
|  |  |  |  |  |  |  |  |  |  |  |  |  |  |  |  |  |  |  |  |  |  |  |
| **11-12** | **–** | **–** | **0,3** | **0,7** | **1,6** | **–** | **–** | **–** | **–** | **–** | **–** | **–** | **–** | **–** | **–** | **–** | **–** | **–** | **–** | **–** | **–** | **–** |
|  | **–** | **–** | **(0,4)** | **(1,0)** | **(1,1)** | **–** | **–** | **–** | **–** | **–** | **–** | **–** | **–** | **–** | **–** | **–** | **–** | **–** | **–** | **–** | **–** | **–** |
|  |  |  |  |  |  |  |  |  |  |  |  |  |  |  |  |  |  |  |  |  |  |  |

**Table S2a**

|  |  | |  | |  | |  | |  | |  | |  | |  | |  | |  | |  | |
| --- | --- | --- | --- | --- | --- | --- | --- | --- | --- | --- | --- | --- | --- | --- | --- | --- | --- | --- | --- | --- | --- | --- |
|  | **MP** | | **RM** | | **1 mm** | | **2 mm** | | **3 mm** | | **4 mm** | | **5 mm** | | **10 mm** | | **RH** | | **LP** | | **LE** | |
|  |  | |  | |  | |  | |  | |  | |  | |  | |  | |  | |  | |
|  | **I** | **II** | **I** | **II** | **I** | **II** | **I** | **II** | **I** | **II** | **I** | **II** | **I** | **II** | **I** | **II** | **I** | **II** | **I** | **II** | **I** | **II** |
|  |  |  |  |  |  |  |  |  |  |  |  |  |  |  |  |  |  |  |  |  |  |  |
|  |  |  |  |  |  |  |  |  |  |  |  |  |  |  |  |  |  |  |  |  |  |  |
| **1 - 2** | **–** | **–** | **–** | **–** | **–** | **0,2** | **–** | **–** | **–** | **2,0** | **–** | **0,8** | **2,6** | **2,0** | **8,2** | **9,0** | **6,4** | **3,8** | **47,2** | **10,0** | **13,4** | **20,8** |
|  | **–** | **–** | **–** | **–** | **–** | **(0,4)** | **–** | **–** | **–** | **1,6** | **–** | **(0,8)** | **2,3** | **(1,6)** | **(6,9)** | **(11,0)** | **(3,8)** | **(1,9)** | **(3,2)** | **(2,8)** | **(9,3)** | **(11,8)** |
|  |  |  |  |  |  |  |  |  |  |  |  |  |  |  |  |  |  |  |  |  |  |  |
| **3 - 4** | **–** | **–** | **0,4** | **0,4** | **4,0** | **3,4** | **1,2** | **3,6** | **10,4** | **10,8** | **32,0** | **15,6** | **28,7** | **15,2** | **49,8** | **23,6** | **64,4** | **56,8** | **50,8** | **63,2** | **80,6** | **37,9** |
|  | **–** | **–** | **(0,5)** | **(0,5)** | **(3,2)** | **(4,2)** | **(1,3)** | **(3,6)** | **(7,2)** | **9,4** | **(8,9)** | **(9,7)** | **2,0** | **(3,2)** | **(13,6)** | **(18,1)** | **(6,3)** | **(3,4)** | **(5,5)** | **(8,6)** | **(5,0)** | **(11,9)** |
|  |  |  |  |  |  |  |  |  |  |  |  |  |  |  |  |  |  |  |  |  |  |  |
| **5 - 6** | **5,6** | **1,6** | **11,4** | **5,4** | **16,2** | **12,4** | **19,4** | **17,8** | **29,8** | **34,0** | **43,3** | **39,2** | **42,9** | **46,0** | **35,2** | **35,8** | **29,0** | **32,4** | **2,0** | **23,8** | **6,0** | **35,5** |
|  | **(1,3)** | **(0,8)** | **(3,1)** | **(2,9)** | **(4,0)** | **(13,2)** | **(3,1)** | **(11,4)** | **(12,6)** | **18,9** | **(4,1)** | **(17,0)** | **3,1** | **(8,1)** | **(15,1)** | **(8,0)** | **(4,7)** | **(2,4)** | **(2,3)** | **(6,5)** | **(5,1)** | **(11,3)** |
|  |  |  |  |  |  |  |  |  |  |  |  |  |  |  |  |  |  |  |  |  |  |  |
| **7 - 8** | **21,6** | **20,5** | **49,4** | **26,4** | **35,2** | **26,0** | **42,5** | **27,4** | **41,6** | **34,0** | **22,3** | **31,0** | **24,0** | **27,6** | **6,4** | **25,2** | **0,2** | **6,2** | **–** | **2,8** | **–** | **5,4** |
|  | **(2,8)** | **(5,3)** | **(5,6)** | **(12,4)** | **(10,3)** | **(3,2)** | **(3,2)** | **(9,7)** | **(7,1)** | **11,9** | **(9,5)** | **(15,7)** | **2,8** | **(5,6)** | **(7,5)** | **(18,1)** | **(0,4)** | **(2,4)** | **–** | **(2,2)** | **–** | **(4,6)** |
|  |  |  |  |  |  |  |  |  |  |  |  |  |  |  |  |  |  |  |  |  |  |  |
| **9 - 10** | **48,3** | **31,4** | **28,8** | **31,1** | **33,2** | **25,4** | **29,7** | **29,4** | **15,0** | **13,0** | **2,2** | **11,0** | **1,8** | **7,4** | **0,4** | **6,0** | **–** | **0,8** | **–** | **0,2** | **–** | **0,4** |
|  | **(3,7)** | **(6,1)** | **(4,6)** | **(5,8)** | **(8,1)** | **(5,2)** | **(4,1)** | **(7,6)** | **(7,8)** | **12,2** | **(1,8)** | **(7,4)** | **1,6** | **(4,9)** | **(0,9)** | **(4,7)** | **–** | **(1,1)** | **–** | **(0,4)** | **–** | **(0,5)** |
|  |  |  |  |  |  |  |  |  |  |  |  |  |  |  |  |  |  |  |  |  |  |  |
| **11 - 12** | **16,7** | **23,8** | **10,0** | **22,6** | **10,6** | **20,4** | **7,2** | **14,0** | **3,2** | **3,8** | **0,2** | **2,2** | **–** | **1,0** | **–** | **0,2** | **–** | **–** | **–** | **–** | **–** | **–** |
|  | **(4,2)** | **(1,7)** | **(6,7)** | **(7,9)** | **(7,3)** | **(7,7)** | **(2,1)** | **(10,6)** | **(4,4)** | **4,4** | **(0,4)** | **(2,6)** | **–** | **(2,2)** | **–** | **(0,4)** | **–** | **–** | **–** | **–** | **–** | **–** |
|  |  |  |  |  |  |  |  |  |  |  |  |  |  |  |  |  |  |  |  |  |  |  |
| **13- 14** | **6,5** | **12,4** | **–** | **9,6** | **0,8** | **6,8** | **–** | **4,2** | **–** | **1,6** | **–** | **0,2** | **–** | **0,8** | **–** | **0,2** | **–** | **–** | **–** | **–** | **–** | **–** |
|  | **(2,7)** | **(4,4)** | **–** | **(7,1)** | **(1,3)** | **(3,0)** | **–** | **(3,5)** | **–** | **2,3** | **–** | **(0,4)** | **–** | **(1,8)** | **–** | **(0,4)** | **–** | **–** | **–** | **–** | **–** | **–** |
|  |  |  |  |  |  |  |  |  |  |  |  |  |  |  |  |  |  |  |  |  |  |  |
| **15 - 16** | **1,0** | **6,8** | **–** | **3,9** | **–** | **2,3** | **–** | **2,2** | **–** | **0,8** | **–** | **–** | **–** | **–** | **–** | **–** | **–** | **–** | **–** | **–** | **–** | **–** |
|  | **(0,8)** | **(4,7)** | **–** | **(3,8)** | **–** | **(1,9)** | **–** | **(2,7)** | **–** | **1,3** | **–** | **–** | **–** | **–** | **–** | **–** | **–** | **–** | **–** | **–** | **–** | **–** |
|  |  |  |  |  |  |  |  |  |  |  |  |  |  |  |  |  |  |  |  |  |  |  |
| **17 - 18** | **0,3** | **3,5** | **–** | **0,6** | **–** | **3,1** | **–** | **1,4** | **–** | **–** | **–** | **–** | **–** | **–** | **–** | **–** | **–** | **–** | **–** | **–** | **–** | **–** |
|  | **(0,4)** | **(2,6)** | **–** | **(0,9)** | **–** | **(2,7)** | **–** | **(0,9)** | **–** | **–** | **–** | **–** | **–** | **–** | **–** | **–** | **–** | **–** | **–** | **–** | **–** | **–** |
|  |  |  |  |  |  |  |  |  |  |  |  |  |  |  |  |  |  |  |  |  |  |  |

**Table S2b**
