# Supplementary material for: Pericentromere clustering in Tradescantia section Rhoeo involves self-associations of AT- and GC-rich heterochromatin fractions, is developmentally regulated, and increases during differentiation
Source: Chromosoma. 2020 Jul 17;129(3):227–42. doi: 10.1007/s00412-020-00740-x (PMC7666280; doi:10.1007/s00412-020-00740-x)
Supplement: Supplementary file 7 — The results of statistical analysis. Out of all the possible pairwise intra-varietal comparisons only 14 [■] showed no significant difference (p > 0.05). The rest of listed comparisons and all the remaining comparisons not listed here disclosed significant difference (p < 0.05) * * As seen in the Table S3, statistically identical were only some adjacent root sectors, which points to a gradual character of the changes in the interphase architecture as the root grows. In line, significant differences appeared as soon as the alternate root sectors were compared. Thus, the used statistics is compatible with the steady reduction of the number of AT- or GC-rich chromatin domains per nucleus during root development (DOC 50 kb). [file 412_2020_740_MOESM5_ESM.doc]

**Table S3.**

|  |  |  |  |  |  |  |  |  |
| --- | --- | --- | --- | --- | --- | --- | --- | --- |
| *tissue type combinations* |  | *ring-forming variety* | | |  | *bivalent-forming variety* | | |
|  |  | *DAPI/AMD* |  | *CMA_3_/DA/DAPI* |  | *DAPI/AMD* |  | *CMA_3_/DA/DAPI* |
|  |  |  |  |  |  |  |  |  |
| *RM versus 1 mm* |  | **■** |  | **■** |  | **■** |  |  |
|  |  |  |  |  |  |  |  |  |
|  |  |  |  |  |  |  |  |  |
| *1 mm vs. 2 mm* |  |  |  | **■** |  |  |  | **■** |
|  |  |  |  |  |  |  |  |  |
| *3 mm vs. 4 mm* |  | **■** |  |  |  |  |  |  |
|  |  |  |  |  |  |  |  |  |
| *4 mm vs. 5 mm* |  | **■** |  |  |  | **■** |  | **■** |
|  |  |  |  |  |  |  |  |  |
|  |  |  |  |  |  |  |  |  |
| *10 mm vs. RH* |  |  |  | **■** |  |  |  |  |
|  |  |  |  |  |  |  |  |  |
| *RH vs. LE* |  |  |  |  |  |  |  | **■** |
|  |  |  |  |  |  |  |  |  |
| *LP vs. LE* |  | **■** |  |  |  | **■** |  | **■** |
|  |  |  |  |  |  |  |  |  |
