# Supplementary material for: Pericentromere clustering in Tradescantia section Rhoeo involves self-associations of AT- and GC-rich heterochromatin fractions, is developmentally regulated, and increases during differentiation
Source: Chromosoma. 2020 Jul 17;129(3):227–42. doi: 10.1007/s00412-020-00740-x (PMC7666280; doi:10.1007/s00412-020-00740-x)
Supplement: Supplementary file 8 — AT-rich domains in ring-forming variety (I) and bivalent-forming variety (II). Frequency (%) of nuclear classes with 1–3 AT-rich domains (DOCX 15 kb). [file 412_2020_740_MOESM6_ESM.docx]

**Table S4.**

|  |  | |  | |  | |  | |  | |  | |  | |  | |  | |  | |  | |
| --- | --- | --- | --- | --- | --- | --- | --- | --- | --- | --- | --- | --- | --- | --- | --- | --- | --- | --- | --- | --- | --- | --- |
|  | **MP** | | **RM** | | **1 mm** | | **2 mm** | | **3 mm** | | **4 mm** | | **5 mm** | | **10 mm** | | **RH** | | **LP** | | **LE** | |
|  |  | |  | |  | |  | |  | |  | |  | |  | |  | |  | |  | |
|  | **I** | **II** | **I** | **II** | **I** | **II** | **I** | **II** | **I** | **II** | **I** | **II** | **I** | **II** | **I** | **II** | **I** | **II** | **I** | **II** | **I** | **II** |
|  |  |  |  |  |  |  |  |  |  |  |  |  |  |  |  |  |  |  |  |  |  |  |
|  |  |  |  |  |  |  |  |  |  |  |  |  |  |  |  |  |  |  |  |  |  |  |
| **1** | **55,6** | **28,5** | **0,2** | **0,1** | **–** | **–** | **–** | **0,5** | **–** | **0,5** | **0,4** | **1,2** | **1,2** | **2,5** | **9,1** | **4,0** | **24,9** | **13,9** | **86,9** | **61,8** | **93,8** | **63,7** |
|  | **(4,9)** | **(7,5)** | **(0,3)** | **(0,2)** | **–** | **–** | **–** | **(0,7)** | **–** | **(0,6)** | **(0,4)** | **(1,0)** | **(1,4)** | **(1,1)** | **(2,4)** | **(1,4)** | **(8,9)** | **(3,9)** | **(4,4)** | **(12,6)** | **(2,5)** | **(6,5)** |
|  |  |  |  |  |  |  |  |  |  |  |  |  |  |  |  |  |  |  |  |  |  |  |
| **2** | **31,3** | **40,7** | **1,2** | **3,0** | **1,0** | **2,7** | **1,6** | **7,0** | **6,2** | **10,4** | **10,3** | **19,6** | **14,1** | **21,7** | **41,7** | **26,1** | **37,7** | **37,6** | **12,5** | **34,0** | **6,2** | **31,6** |
|  | **(1,8)** | **(5,8)** | **(1,2)** | **(2,4)** | **(1,2)** | **(2,0)** | **(0,4)** | **(6,6)** | **(2,9)** | **(3,7)** | **(5,9)** | **(7,2)** | **(6,7)** | **(6,7)** | **(5,8)** | **(1,6)** | **(4,2)** | **(4,8)** | **(3,7)** | **(12,0)** | **(2,5)** | **(4,0)** |
|  |  |  |  |  |  |  |  |  |  |  |  |  |  |  |  |  |  |  |  |  |  |  |
| **3** | **10,0** | **22,8** | **4,3** | **13,9** | **5,0** | **14,6** | **9,2** | **23,4** | **21,6** | **30,3** | **26,8** | **30,7** | **31,4** | **38,6** | **25,9** | **39,4** | **29,5** | **36,8** | **0,6** | **4,2** | **–** | **4,7** |
|  | **(2,1)** | **(6,4)** | **(1,8)** | **(11,6)** | **(1,2)** | **(8,2)** | **(2,5)** | **(15,9)** | **(9,1)** | **(7,1)** | **(6,7)** | **(4,2)** | **(4,6)** | **(7,9)** | **(5,2)** | **(5,2)** | **(5,3)** | **(7,7)** | **(0,8)** | **(2,4)** | **–** | **(3,2)** |
|  |  |  |  |  |  |  |  |  |  |  |  |  |  |  |  |  |  |  |  |  |  |  |
| **1 - 3** | **96,9** | **92,0** | **5,7** | **17** | **5,9** | **17,3** | **10,8** | **30,9** | **27,8** | **41,2** | **37,5** | **51,5** | **46,7** | **62,8** | **76,7** | **69,5** | **92,1** | **88,3** | **100,0** | **100,0** | **100,0** | **100,0** |
|  | **(2,9)** | **(5,0)** | **(2,9)** | **(13,0)** | **(0,5)** | **(10,2)** | **(2,2)** | **(23,1)** | **(10,8)** | **(10,9)** | **(12,3)** | **(10,2)** | **(9,9)** | **(12,4)** | **(4,5)** | **(6,5)** | **(1,7)** | **(3,0)** | **(0,0)** | **(0,0)** | **(0,0)** | **(0,0)** |
|  |  |  |  |  |  |  |  |  |  |  |  |  |  |  |  |  |  |  |  |  |  |  |
